# Melanoma causes phenotypic modulations and metabolic switches of iNKT cells influencing clinical outcomes

**DOI:** 10.3389/fimmu.2025.1703754

**Published:** 2026-01-05

**Authors:** Emmanuelle Degeorges, Stéphane Mouret, Pauline Girard, Camille Niveau, Mélanie Cettour-Cave, Eleonora Sosa Cuevas, Florence De Fraipont, Julie Charles, Philippe Saas, Caroline Aspord

**Affiliations:** 1 Institute for Advanced Biosciences, Team: Cell Dynamics, Immunity, Metabolism and Cancer, Inserm U1209, CNRS UMR5309, Univ. Grenoble Alpes, Grenoble, France; 2 Etablissement Français du Sang Auvergne-Rhône-Alpes, R&D Laboratory, Grenoble, France; 3 Univ. Grenoble Alpes, Dermatology, Allergology & Photobiology Department, CHU Grenoble Alpes, Grenoble, France; 4 Medical Unit of Molecular Genetic (Hereditary Diseases and Oncology), Grenoble University Hospital, Grenoble, France

**Keywords:** iNKT cells, melanoma, immunometabolism, immune checkpoint, clinical outcome

## Abstract

**Introduction:**

Invariant NKT (iNKT) cells are crucial effectors in cancer immunosurveillance, due to their immunomodulatory potential through a broad range of effector and regulatory functions. Yet, their use as targets or vectors for cancer immunotherapy in cancer yielded inconsistent outcomes, due to potential tumor immune escape mechanisms. Limited information is available regarding the potential dysfunctions of iNKT cells in melanoma patients, and their clinical significance. A better understanding of iNKT cell biology and subversion in these patients would help designing new immunotherapies and improving clinical translations.

**Methods:**

Here, we depicted extensive phenotypic, metabolic and functional features of circulating and tumor-infiltrating iNKT cells in melanoma patients, and assessed their clinical relevance.

**Results:**

We observed that iNKT cells infiltrated melanoma tumors in a gender- and site-dependent manner, and were associated with poor clinical outcome. Invariant NKT cells exhibited a higher basal activation status together with a skewed expression of NK receptors (NKR), NKG2 and immune checkpoints (ICP), as well as a shift toward regulatory iNKT (iNKTreg) cell profile in the melanoma microenvironment. We identified LAG3, CTLA4 and TIM3 as critical negative prognosis factors of clinical evolution. Moreover, tumor-infiltrating iNKT cells displayed a dampened metabolic activity with a decreased glycolysis dependency; such perturbed energetic metabolism impacted patient clinical outcome. Furthermore, iNKT cells revealed distinct metabolic profiles depending on their activation status and ICP profile, underlining critical connections between iNKT cell features and metabolic pattern.

**Discussion:**

Overall, our study reveals major phenotypic and metabolic disturbances of circulating and tumor-infiltrating iNKT cells in melanoma, with clinical impacts. By unveiling new key features and skewing details on iNKT cells in melanoma, our study paves the way for innovative combination strategies exploiting metabolic pathways and/or disturbed ICP profiles to overcome immune subversion and better harness the potential of iNKT cells for cancer immunotherapy.

## 1 Introduction

Despite recent improvements in metastatic melanoma treatment using targeted therapies or immunomodulatory strategies, the long-term control of the tumor in a majority of patients still remains a challenge (1). A better understanding of the tumor immune escape mechanisms is crucial to design new therapeutic strategies and potentiate existing immunomodulatory therapies to achieve better clinical success.

The tumor microenvironment (TME) comprises many immune cell types (2, 3), including natural killer T (NKT) cells that are still not fully explored despite their fascinating properties. Within the innate immunity, numerous cell types exist, including several subsets of innate lymphoid cells (ILCs), comprising NK cells (4), and innate-like T (ILT) cells including NKT cells. NKT cells represent less than 0.1% of circulating T cells, but display potent properties and have unique contributions to many types of immune responses (5–7). NKT cells are composed of the type I and II NKT subsets. Type I NKT cells, also called invariant NKT (iNKT) cells, are characterized by the simultaneous expression of NK lineage receptors and invariant T-cell receptors (TCRs) consisting of the Vα24-Jα18 chain paired mainly with Vβ11 in humans. In contrast, type II NKT cells exhibit a more diverse TCR repertoire. iNKT cells have the unique ability to recognize foreign or self-glycolipid antigens presented in the context of the non-polymorphic major histocompatibility complex (MHC) class I-like molecule CD1d. The alpha-galactosylceramide (αGalCer), a glycosphingolipid extracted from marine sponges, is the prototype ligand recognized by iNKT cells and is used in iNKT cell-based immunotherapies. At the steady state, iNKT cells are mainly found in the liver, the spleen, and adipose tissue, and less in the lungs, intestines, and lymph nodes. These iNKT cells uniquely combine the features of both innate and adaptive immunity, displaying a quick response to antigen exposure and exhibiting regulatory or cytotoxic functions (5, 7). Thus, iNKT cells are endowed with a profound immunomodulatory potential through a broad range of immune effector and regulatory functions, bridging the innate and adaptive immune systems and enhancing or dampening the immune responses (7–9). Thus, iNKT cells may influence the initiation and regulation of the immune responses. Upon activation through lipid antigen/CD1d complexes or cytokines, iNKT cells proliferate, exhibit cytotoxic activity, and rapidly secrete a large variety of pro-inflammatory (e.g., IFNγ, TNFα, and IL-17) and immunoregulatory (e.g., IL-4 and IL-10) cytokines. They exhibit diverse profiles associated with multiple functional subsets [NKT1, NKT2, NKT10, NKT17, and FoxP3^+^ regulatory NKT cells (NKTregs)] (10). Once activated, iNKT cells have the ability to recruit, activate, and modulate other immune cells through the release of chemokines/cytokines or through cell contact-dependent signals (via NK activating receptors).

In cancer immunosurveillance, alongside other cell types, in particular NK cells (11), iNKT cells are crucial effectors. Due to their immunoregulatory and cytotoxic properties, they orchestrate the innate and adaptive immune responses against tumors (10, 12–15). iNKT can sense changes in the lipid metabolism of cancer cells and directly kill tumor cells through CD1d-mediated antigen recognition or target ligands on tumor or stressed cells through the expression of various NK activating receptors (e.g., NKG2D, NKp30, and NKp44) or tumor necrosis factor (TNF) receptors, subsequently driving cell death pathways through the release of cytotoxic granules (5, 7). iNKT can also favor the antitumor responses by regulating suppressive cells [e.g., myeloid-derived suppressive cells (MDSCs)], influencing the polarization of tumor-associated macrophages (TAMs), trans-activating NK cells, enhancing the cytotoxic potential of cytotoxic T lymphocytes (CTLs), and stimulating the maturation of B cells (10, 16). Concerning dendritic cells (DCs), iNKT cells promote their maturation and enhance their cross-presentation activity, ultimately triggering antitumor CD8 T-cell responses (17).

All these unique and crucial antitumor properties together with their functional plasticity render iNKT cells attractive both as targets or vectors for cancer immunotherapy (8, 9, 12, 16). Several iNKT-based therapeutic approaches have been developed. The administration of iNKT agonists such as αGalCer or αGalCer-pulsed DCs has demonstrated effective killing against tumors in both mouse models and patients through robust proliferation of iNKT cells and enhanced antigen-specific expansion of CD8 T cells (18). The adoptive transfer of *ex vivo*-expanded iNKT cells can inhibit tumor growth in tumor-bearing humanized mice (19) and is feasible and safe in patients (20). The generation of chimeric antigen receptor (CAR) iNKT cells has also demonstrated notable antitumor efficacy (21). However, clinical trials in patients with cancer have yielded inconsistent outcomes due to potential tumor immune escape mechanisms (8, 22). Failure to respond to iNKT cell-based immunotherapies may be linked to an impaired iNKT cell function due to an immunosuppressive TME. Better understanding of the iNKT cell biology and subversion in patients with cancer would help in the design of new immunotherapies and improve clinical translation.

In patients with cancer, alterations in the iNKT cell number and functionality have been highlighted in several cancer types, including reduced frequency, proliferative and functional defects, and a shift toward the type 2 phenotype (10, 16). Moreover, iNKT cells have been identified within tumor-infiltrating cells in multiple cancers (16). However, their clinical relevance remains mostly unknown. Regarding melanoma, a few studies have reported functional impairments of peripheral iNKT cells in patients with melanoma compared with healthy controls. In a B16 mouse melanoma model, iNKT cells infiltrated tumors, but displayed dysfunctional states with increased expression of the exhaustion markers (including PD1, CTLA4, and TIM3) and of NKG2D, as well as low levels of IFNγ production associated with IL-4, IL-10, and IL-17 production within the TME (23).

Immunometabolism emerges as critical in the regulation of the immune cell functions in cancer (24–27). Indeed, the energetic profile and metabolic pathways determine the cell activation, differentiation, and functions (28, 29). Cells use two main pathways—oxidative phosphorylation (OXPHOS) and aerobic glycolysis—to produce ATP, mostly governed by the AMP-activated protein kinase (AMPK) and mechanistic target of rapamycin (mTOR) signaling pathways, respectively. Upon activation, immune cells undergo a metabolic switch from OXPHOS to glycolysis, which provides essential resources to support new protein synthesis for cell proliferation, activation, and function. Metabolism is critical in driving distinct differentiation programs, and metabolic reprogramming shapes the immune cell differentiation and function (30). An impaired metabolism has been reported to cause the dysfunction of antitumor immune cells in the TME (31). Tumor cells undergo a metabolic switch from OXPHOS to glycolysis to support active proliferation, creating an environment hostile to infiltrating immune cells, with competition for limited nutrients, oxygen changes, and the accumulation of toxic metabolites in the TME that impair the immune responses (24–26). This suppressive metabolic microenvironment supports the metabolism of tumor-promoting cells [CD4^+^ regulatory T cells (Tregs), MDSCs, and tolerogenic DCs] and dampens the immune cell proliferation, survival, and functions, contributing to ineffective immune responses and cancer progression (24). Thus, the metabolic reprogramming of immune cells within the TME could be responsible for immune subversion and tumor immune escape. However, the metabolic features and perturbations of immune cells—in particular iNKT cells—in melanoma remain largely unknown. A few studies revealed that metabolism controls the molecular processes of NKT cells to respond to lipid antigens and subsequent functions (32, 33). In tumor-bearing mice, circulating and tumor-infiltrating NKT cells exhibited an unbalanced metabolism characterized by a suppressed glucose metabolism (as attested by a decreased expression of the transporters Glut1 and Glut3 and of the glycolysis-related enzymes) (23). In addition, the genes coding the key enzymes involved in cholesterol synthesis (i.e., *Hmgcs1* and *Idl1*) were downregulated in circulating and tumor-infiltrating NKT cells.

There is very limited information on the phenotypic and metabolic features and the potential dysfunctions of iNKT cells in patients with melanoma, as well as their clinical significance. Dissecting the fate and potential skewing of iNKT cells in the TME is challenging, but is critical for harnessing iNKT cells for cancer immunotherapy and for the development of effective antitumor therapies. This prompted us to explore the detailed status of iNKT cells in patients with melanoma in relation to disease outcomes. The aim of this study was to better understand the physiopathology and potential hijacking of iNKT cells in melanoma by depicting the iNKT cell features together with their clinical significance. We investigated the phenotype (i.e., activation status, ICP expression, and NK receptor profile) and metabolic fitness of iNKT cells in the blood and tumor of patients with melanoma in comparison to healthy controls and non-tumoral tissues and assessed their clinical relevance. This study highlights crucial iNKT cell features/dysfunctions and promising potential biomarkers for clinical evolution in melanoma. Such understanding may help harness the power of iNKT cells against tumors and allow improving cancer immunotherapies and patient outcomes.

## 2 Materials and methods

### 2.1 Melanoma patients and healthy donor samples

All procedures were approved by the Ethics Committee of Grenoble University Hospital (CHUGA) and the French Blood Agency’s Institutional Review Board Committee (IRB) and accredited by the Ministry of Education and Research under reference no. AC-2020–3959 (EFS) and collection no. AC-2014-2094 (CHUGA). Mandatory written informed consent was obtained from all donors prior to their participation in this study, and samples were analyzed anonymously. Blood samples were obtained from healthy donors (HDs; *n* = 38) and from stage I–IV melanoma patients (*n* = 48). Primary tumors and lymph node or cutaneous metastatic tumors (*n* = 49) were obtained from patients with melanoma. Non-tumoral tissues (tonsils; *n* = 9) were also obtained from volunteers who underwent tonsillectomy due to recurring angina. The clinical features of the patients with melanoma are shown in **Supplementary Tables S1** and **S2**. Progression-free survival (PFS) and overall survival (OS) were calculated both from the diagnosis and the sampling time.

### 2.2 PBMC isolation and tumor-infiltrating/tonsil-derived immune cell isolation

Peripheral blood mononuclear cells (PBMCs) were obtained by Ficoll density gradient separation (Eurobio, Les Ulis, France) of the blood from HDs and patients with melanoma. The cells were frozen and stored at −150°C until use. Tumor samples and non-tumoral tissues were cut into small fragments using a sterile scalpel and enzymatically digested with collagenase D (final concentration, 2.5 mg/ml) and DNase I (final concentration, 100 μg/ml) (Sigma, St. Louis, MO, USA) for 30 min at 37°C with 5% of CO_2_, followed by mechanical disruption. Cell suspensions were obtained by filtering the mixture through a 50-μm sieve with RPMI 10% fetal bovine serum (FBS), frozen, and stored at −150°C before use. After thawing, tumor-infiltrating cells were first cleared of tumor cells using an adhesion step in a 75-cm^2^ flask.

### 2.3 Assessment of the proportion, activation status, ICP expression, and killer cell lectin-like receptor/natural cytotoxicity receptor profiles of iNKT cells by multiparametric flow cytometry

PBMC or tissue-derived immune cell suspensions were stained in phosphate-buffered saline (PBS) 2% fetal calf serum (FCS) to define the iNKT cells and to depict their activation status, ICP expression, and killer cell lectin-like receptor (KLR)/natural cytotoxicity receptor (NCR) profiles. To depict iNKT cells, the following antibodies were used: BV570 anti-CD45 (BioLegend, Paris, France), APC-H7 anti-CD3 (BD Biosciences, Le Pont de Caix, France; hereinafter abbreviated as BD), and PE-Cy7 or APC anti-iNKT (BioLegend, Paris, France), as well as Live/Dead PE-Texas Red (Thermo Fisher, Massy, France). To assess their basal activation status, we additionally used the following antibodies: PE-Cy5 or PerCP anti-CD69, PE-Cy7 anti-CD86 (BD, Paris, France), PE anti-CD40 (Beckman, Villepinte, France), and APC anti-CD25 (Thermo Fisher, Massy, France). The ICPs were analyzed using BV650 anti-PD1, PE anti-OX40L, PerCP-Cy5.5 anti-PDL2, PE anti-41BB, PE anti-41BBL, PE-Cy7 anti-OX40, PE-Cy7 anti-PDL1, PerCP Cy5.5 anti-PD1, PerCP Cy5.5 anti-ICOS (BD, Paris, France), APC anti-LAG3, and APC anti-ICOSL antibodies (Thermo Fisher, Massy, France). The activating and inhibitory natural killer cell receptor (NKR) and KLR profiles were depicted using the antibodies PE-Cy7 anti-NKG2D, PE anti-NKp30 (BD, Paris, France), PC7 anti-NKp46 (Beckman, Villepinte, France), PE anti-NKG2A, PerCP anti-NKG2C (BioTechne, Minneapolis, MN, USA), and APC anti-NKp44 (Thermo Fisher, Massy, France). Their differentiation stage was assessed by labeling with PE anti-CD27 and PE-Cy7 anti-CD45RA antibodies (BD, Paris, France). Regulatory iNKT cells were evaluated using FoxP3 intranuclear labeling (eBiosciences, Paris, France), while cytotoxic iNKT cells were assessed using APC anti-CD56 antibodies. Labeling with the corresponding control isotypes was performed to set the threshold of expression of the activation markers, ICP, NKR, KLR, and FoxP3. After washing, the cell suspensions were finally resuspended in BD FACS lysing solution, acquired using a FACS LSRII flow cytometer (BD, Paris, France), and analyzed using BD FACS DIVA 9 software (version 9.0.1). To ensure quality control during the study, we performed a systematic standardization of the fluorescence intensities using Cytometer Setup and Tracking (CST) beads (BD, Paris, France). The numbers of iNKT events acquired per sample were as follows: for HDs, median = 824, range = 13–2150; for patient blood, median = 114, range = 11–407; for patient tumor, median = 38, range = 14–1,026; and for tonsils, median = 68, range = 36–82. Samples with less than 10 events in the iNKT cell gate were excluded from the analysis.

### 2.4 Metabolism profiling using the single-cell energetic metabolism by profiling translation inhibition method

To study the cell metabolism, up to 2 × 10^6^ PBMCs or tumor-infiltrating immune cells were cultured in 96-well plates in RPMI 1640 GLUTAMAX I supplemented with gentamicin (20 µg/ml), non-essential amino acids (MEM 1×; Invitrogen, Carlsbad, CA, USA), sodium pyruvate (1 mM; Sigma, St. Louis, MO, USA; complete RPMI), and 10% heat-inactivated FCS in a humidified incubator maintained at 37°C with 5% CO_2_ atmosphere. The cells were treated for 30 min with dimethyl sulfoxide (DMSO; control), 2-deoxy-d-glucose (DG; final concentration, 100 mM) (Sigma, St. Louis, MO, USA), oligomycin (Oligo; final concentration, 1 mM) (Ozyme, Saint-Cyr-l’École, France), or a combination of these drugs (at the final concentrations mentioned previously). Puromycin (Puro; final concentration, 10 µg/ml) (Cayla, Toulouse, France) was added for 30 min after the metabolic inhibitor treatment. The cells were then washed in cold PBS and stained with the fluorescent cell viability marker and primary conjugated antibodies against the desired surface markers for 20 min at 4°C in PBS. After washing, the cells were fixed and permeabilized using FOXP3 Fixation and Permeabilization Buffer (Thermo Fisher, Massy, France) following the manufacturer’s instructions. Intracellular staining of Puro was performed by incubating the cells for 30 min at 4°C in a permeabilization buffer with Alexa Fluor 488 anti-Puro monoclonal antibody (Merck, Lyon, France).

### 2.5 Statistical analysis

Statistical analyses were performed with the GraphPad Prism Software (version 9.3.1) using the non-parametric unpaired Mann–Whitney test, unpaired Kruskal–Wallis test followed by Dunn’s multiple comparison posttest, or two-way repeated measures ANOVA, or the mixed-effects model followed by Sidak’s multiple comparison posttest, or the log-rank test. The significance threshold was set at *p* < 0.05. Survival analyses were performed (Kaplan–Meier followed by correction for multiple comparisons) and heat maps were constructed using the survival (3.3.1), survminer (0.4.9), gplots (3.1.3), and RColorBrewer (1.1.3) packages with the RStudio software R version 4.4.1.

## 3 Results

### 3.1 High proportions of tumor-infiltrating iNKT cells are associated with poor clinical outcomes in metastatic melanoma patients

**Figure 1:**
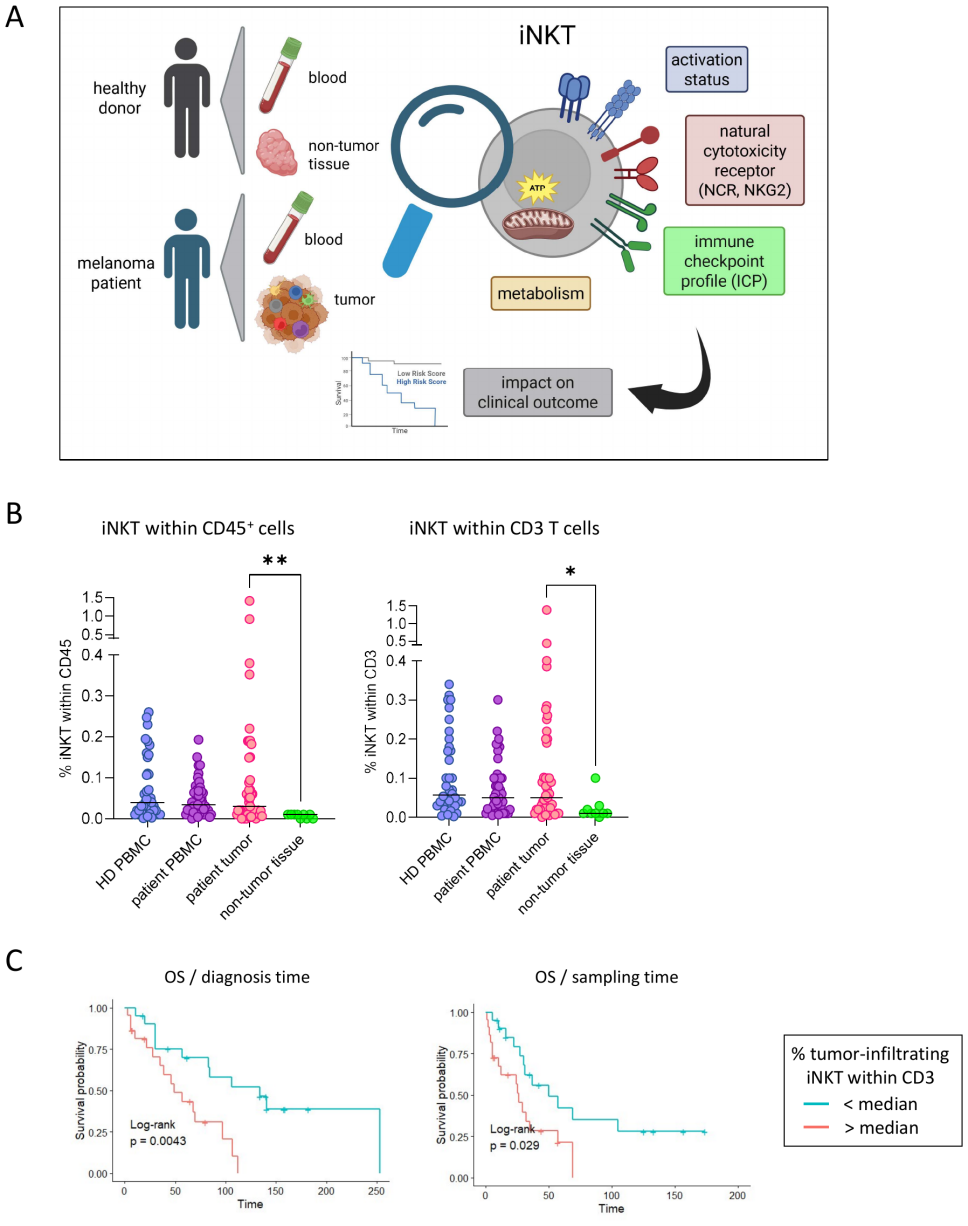
Invariant natural killer T (iNKT) cells infiltrate melanoma tumors and are associated with worse prognosis. **(A)** Experimental design. Peripheral blood mononuclear cells (PBMCs) and tonsil-derived cell suspensions from healthy donors (HDs) and PBMCs and tumor-immune cell infiltrates from melanoma patients were submitted to flow cytometry analysis to depict the following features of iNKT cells: proportion/frequency, activation status, killer cell lectin-like receptor (KLR), natural cytotoxicity receptor (NCR) expression, immune checkpoint (ICP) profile, and metabolism. Ultimately, the impact of the features of iNKT cells on the patients’ clinical outcomes was evaluated. Created using BioRender. **(B)** Proportions of iNKT cells within CD45^+^ cells (*left panel*) and CD3^+^ T cells (*right panel*) in PBMCs (*n* = 38) and tonsil-derived cell suspensions (*n* = 9) from HDs and in PBMCs (*n* = 47) and tumor-immune infiltrates (*n* = 46) from melanoma patients. *Bars* indicate the median. Comparisons were performed using the Kruskal–Wallis test with Dunn’s multiple comparison posttest. *p < 0.05, **p < 0.01. **(C)** Comparative overall survival (OS) of patients according to the proportions of tumor-infiltrating iNKT cells. OS was calculated from the diagnosis time (*left panel*) or from the sampling time (*right panel*). Groups were separated according to the median percentage of iNKT cells within CD3^+^ T cells (high, *red*; low, *blue*; *n* = 43 in total, 21–22 per group). Log-rank test. Only significant statistics are displayed on the graphs.

**Figure 2:**
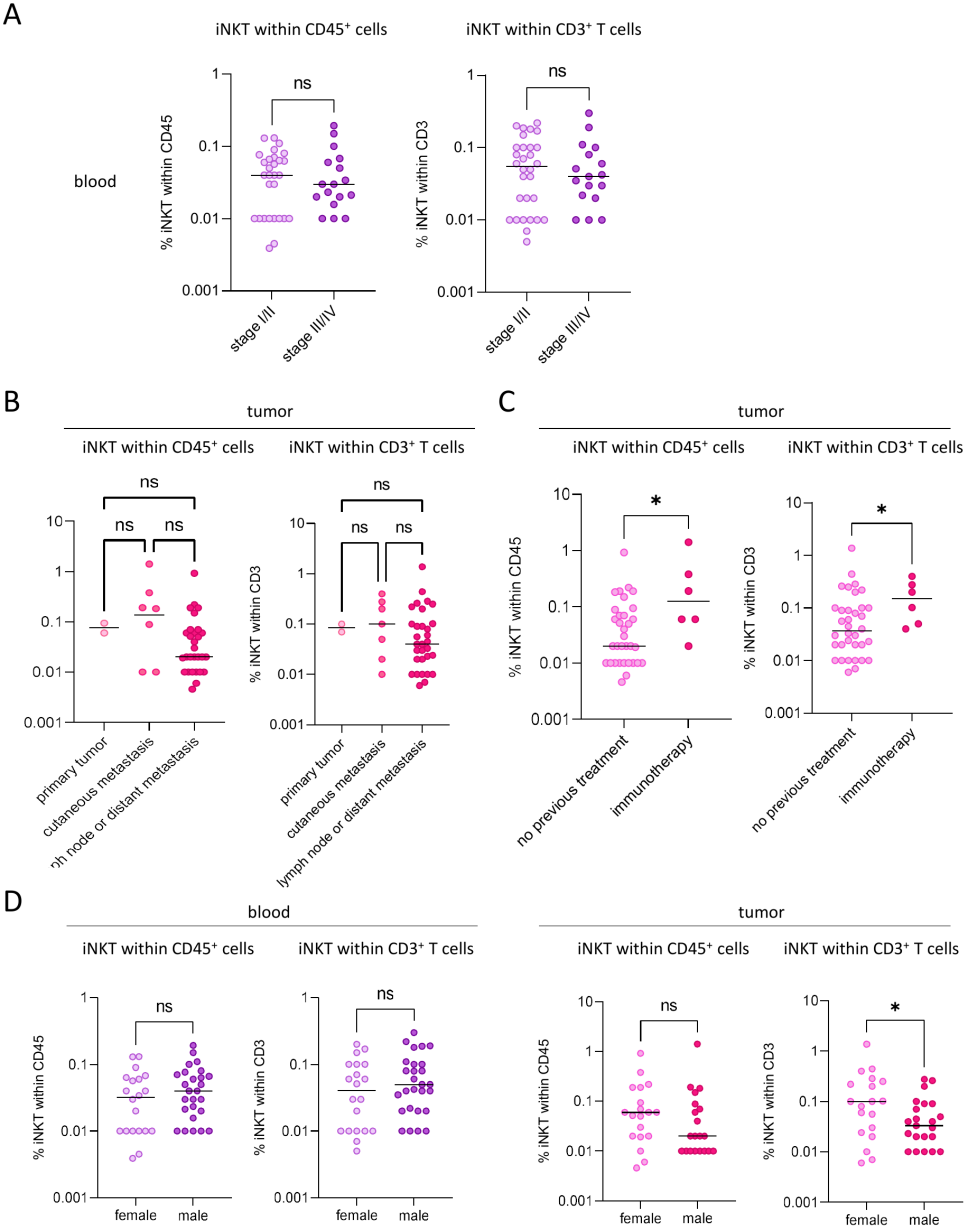
Impact of the clinical parameters on the circulating and tumor-infiltrating invariant natural killer T (iNKT) cell frequencies in melanoma patients. Peripheral blood mononuclear cells (PBMCs) and tumor immune infiltrates from melanoma patients were submitted to flow cytometry analysis to depict the proportions of iNKT cells in the blood and tumor. Comparisons were performed according to the clinical parameters of patients: disease stage, treatment before sampling, tumor localization, and gender. **(A)** Comparative proportions of iNKT cells in the blood of melanoma patients according to the disease stage at sampling time: blood early stage I/II (*n* = 30) and blood advanced stage III/IV (*n* = 17). *P*-values were calculated using unpaired non-parametric Mann–Whitney test. **(B)** Comparative proportions of iNKT cells in the tumor of melanoma patients according to tumor localization: primary tumor (*n* = 2), cutaneous metastasis (*n* = 7), and lymph node or distant metastasis (*n* = 32). *P*-values were calculated using non parametric Kruskal–Wallis test followed by Dunn’s multiple comparison test. **(C)** Comparative proportions of iNKT cells in the tumor of melanoma patients according to treatment before sampling: untreated/surgery (*n* = 38) and immunotherapy (*n* = 6). *P*-values were calculated using unpaired non-parametric Mann–Whitney test. **(D)** Comparative proportions of iNKT cells in the blood (*left panels*) and tumor (*right panels*) of melanoma patients according to gender: blood (F: *n* = 20, M: *n* = 27) and tumor (F: *n* = 19, M: *n* = 23). *P*-values were calculated using unpaired non-parametric Mann–Whitney test. * p<0.05; ns, non significant.

We first evaluated the proportions of iNKT cells in the blood and tumor samples of patients with melanoma compared with the blood of HDs and non-tumoral tissue (**Figure 1A**, **Supplementary Figure S1**). As the majority of the tumor samples from patients were metastatic lymph nodes, we used tonsils as the control for the lymph node tissues of the patients, which were the closest available control tissue. Whereas the proportions of circulating iNKT cells were similar between HDs (mean = 0.11%/median = 0.06% of CD3^+^ T cells) and patients (mean = 0.07%/median = 0.05% of CD3^+^ T cells), we observed that iNKT cells accumulated within the melanoma tumors (mean = 0.43%/median = 0.05% of CD3^+^ T cells) compared with non-tumoral tissues (mean = 0.02%/median = 0.01% of CD3^+^ T cells) (**Figure 1B**, **Supplementary Figure S1A**). As the range of the iNKT cell frequencies was highly heterogeneous between patients, we explored whether clinical parameters could influence the proportions of circulating and tumor-infiltrating iNKT cells. When classified according to disease stage, patients with early stage I–II displayed similar frequencies of circulating iNKT cells compared to patients with advanced stage III–IV melanoma (**Figure 2A**). Interestingly, we noticed a trend toward a higher frequency of iNKT cells (within CD45^+^ cells) in cutaneous metastases compared with lymph node metastases (**Figure 2B**), although not significant, which suggests that tumor localization can influence the iNKT cell frequency. When classified according to previous treatment before tumor sampling, patients who received immunotherapy (e.g., IFNα, DC vaccine, or ICP blocker) displayed significantly higher proportions of iNKT cells (among CD45^+^ cells or CD3^+^ T cells) infiltrating the tumor (**Figure 2C**), revealing that immunotherapy may enhance iNKT cell recruitment to the tumor site. Moreover, despite similar proportions of iNKT cells in patient blood between men and women (**Figure 2D, left**), we observed higher proportions of iNKT cells among CD3^+^ T cells infiltrating the tumor in women compared with men (**Figure 2D, right**), suggesting that gender may dictate the level of tumor-infiltrating iNKT cells. Strikingly, we found that a higher proportion of tumor-infiltrating iNKT cells predicted worse clinical outcomes as it was linked with a shorter OS (**Figure 1C**, **Supplementary Table S3**). Altogether, these data highlight that iNKT cells infiltrate melanoma tumors in a gender- and site-dependent manner and are associated with poor clinical outcomes.

### 3.2 Circulating and tumor-infiltrating iNKT cells from melanoma patients displayed an altered activation status with perturbed expression of NCR/KLR and a modified immune checkpoint profile

**Figure 3:**
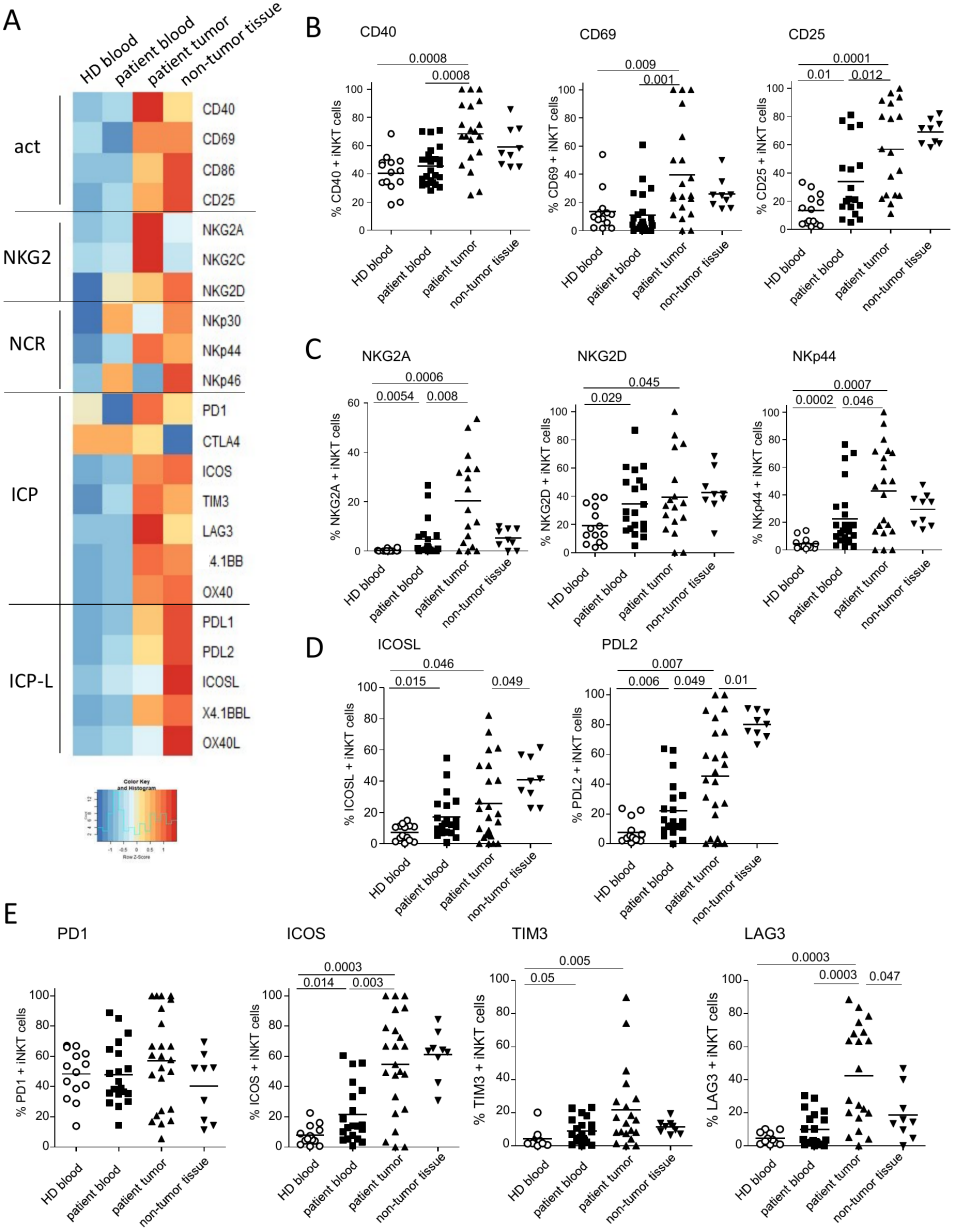
Circulating and tumor-infiltrating invariant natural killer T (iNKT) cells from patients exhibit perturbations in their phenotypic features. Peripheral blood mononuclear cells (PBMCs; *n* = 14) and tonsil-derived cell suspensions (*n* = 9) from healthy donors (HDs) and PBMCs (*n* = 19–24) and tumor immune infiltrates (*n* = 17–24) from melanoma patients were submitted to flow cytometry analysis to depict their activation status, killer cell lectin-like receptor (KLR) and natural cytotoxicity receptor (NCR) expression, and immune checkpoint (ICP) and ICP ligand (ICP-L) profiles. **(A)** Heat map based on the median proportions of marker-expressing iNKT cells in the different groups. *Each column* represents the median value of the parameters from all individuals of the corresponding group. Color code by line. **(B)** Comparative proportions of iNKT cells expressing the activation markers CD40, CD69, and CD25 in the different groups. **(C)** Comparative proportions of iNKT cells expressing NKG2A, NKG2D, and NKp44 in the different groups. **(D)** Comparative proportions of iNKT cells expressing ICOSL and PDL2 in the different groups. **(E)** Comparative proportions of iNKT cells expressing PD1, ICOS, TIM3, and LAG3 in the different groups. *P*-values were calculated using the non-parametric unpaired Mann–Whitney test. Only significant statistics are displayed on the graphs.

We further examined the features of circulating and tumor-infiltrating iNKT cells in patients with melanoma. To gain insights into the impact of melanoma on iNKT cells, we assessed their differentiation stage, their activation status, their expression of NCR/KLR, and the ICP molecules (**Figure 3**, **Supplementary Figures S1B–F**; **S2**), as these molecules determine the fate of iNKT cells and orientate their potentialities to interact with target cells and subsequently modulate other immune cells.

The heat map representation of all phenotypic features of iNKT cells highlighted the differences between groups and revealed clear specific features of each group (**Figure 3A**). We first investigated the basal activation status of iNKT cells by analyzing the markers shared by both antigen-presenting cells (CD40 and CD86) and T cells (CD25 and CD69) (**Figure 3B**, **Supplementary Figure S2A**). We observed higher proportions of circulating CD25-expressing iNKT cells in patients compared with HDs (**Figure 3B**) and higher proportions of activated tumor-infiltrating iNKT cells compared with circulating iNKT cells in patients or HDs, although no differences were observed between tumors and non-tumoral tissues (**Figure 3B**, **Supplementary Figure S2A**). We next explored the NCR/KLR expression profiles (**Figure 3C**, **Supplementary Figure S2B**). We observed that the circulating iNKT cells from patients with melanoma exhibited higher levels of NKG2A, NKG2D, and NKp44 compared with those from HDs, and tumor-infiltrating iNKT cells expressed more NKG2A, NKG2C, and NKp44 compared with the blood of patients and HDs (**Figure 3C**, **Supplementary Figure S2B**).

We then assessed the ICP expression profiles, i.e., both ICP ligands (ICP-L) (**Figure 3D**, **Supplementary Figure S2C**) and receptors (**Figure 3E**, **Supplementary Figure S2D**). The expression of ICP by iNKT cells in patients was highly heterogeneous, particularly for tumor-infiltrating iNKT cells. We observed higher proportions of circulating iNKT cells expressing ICOSL, PD-L2 (**Figure 3D**), ICOS, TIM3 (**Figure 3E**), and 4-1BB (**Supplementary Figure S2D**) in melanoma patients compared with HDs. Whereas the proportions of ICP-L-expressing iNKT cells (ICOSL, PDL2, and OX40L) (**Figure 3D**, **Supplementary Figure S2C**) decreased in tumors compared with non-tumoral tissues, we observed that LAG3 was the only ICP whose expression was highly heightened on tumor-infiltrating iNKT cells compared with non-tumoral tissues (**Figure 3E**). In addition, the proportions of ICOS^+^, LAG3^+^, 4-1BB^+^, and OX40^+^ tumor-infiltrating iNKT cells were higher compared with circulating iNKT cells from patients or HDs (**Figure 3E**, **Supplementary Figure S2D**).

We further assessed the differentiation stage based on the expression of CD45RA and CD27, allowing to distinguish naive (CD45RA^+^CD27^+^) and central memory (CM; CD45RA^−^CD27^+^) cells that are not yet differentiated from the effector memory (EM; CD45RA^−^CD27^−^) and terminally differentiated (EMRA; CD45RA^+^CD27^−^) phenotypes that display effector functions (**Supplementary Figure S2E**). We observed decreased proportions of circulating iNKT cells in the CM stage in patients compared with HDs and a decreased naive state concomitant to an increased CM status in tumors compared with non-tumoral tissues. This suggests a higher differentiation of iNKT cells from the naive to the CM stage in tumors. Notably, when evaluating the regulatory phenotype of iNKT cells using the CD25 marker and the FoxP3 transcription factor, we observed increased levels of CD25^hi^FoxP3^+^ iNKTreg cells in the blood and tumor of patients with melanoma compared with circulating iNKT cells in HDs (**Supplementary Figure S2F**).

Altogether, these data highlight that iNKT cells display a higher basal activation status together with a skewed expression of NKR, KLR, and ICPs and a shift toward an iNKTreg profile in the melanoma microenvironment.

### 3.3 Invariant NKT cell phenotypic features dictate clinical outcomes, with LAG3 being the strongest negative prognostic factor for clinical evolution

**Figure 4:**
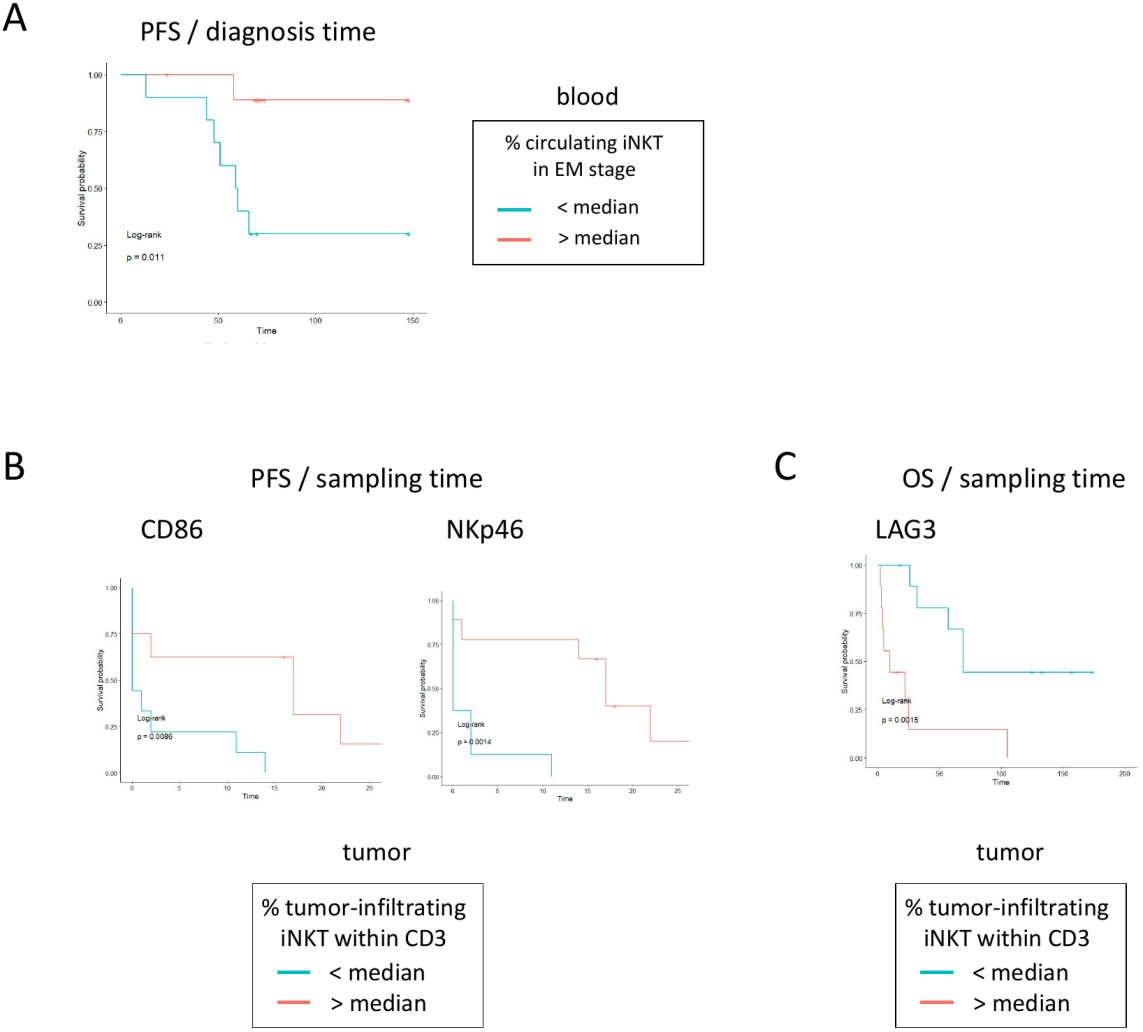
Differentiation state and phenotypic profiles of invariant natural killer T (iNKT) cells dictate the clinical outcomes of melanoma patients. Peripheral blood mononuclear cells (PBMCs; *n* = 18–24) and tumor immune cell infiltrates (*n* = 18–24) from melanoma patients were submitted to flow cytometry analysis to depict their activation status, killer cell lectin-like receptor (KLR) and natural cytotoxicity receptor (NCR) expression, immune checkpoint (ICP) and ICP ligand (ICP-L) profiles. The impact of the phenotypic features of iNKT cells on clinical evolution was then evaluated using Kaplan–Meier analyses. **(A)** Comparative progression-free survival (PFS) of patients [from the diagnosis time (*left panel*) or from the sampling time (*right panel*)] according to the effector memory (EM) status of circulating iNKT cells. Groups were separated using the median percentage of iNKT cells in the EM stage (*n* = 20 in total, 10/group). **(B)** Comparative PFS of patients (from the sampling time) according to the proportion of tumor-infiltrating iNKT cells expressing CD86 or NKp46. Groups were separated using the median percentage of the corresponding marker (*n* = 18 and 19, respectively, in total, 7–10/group). **(C)** Comparative overall survival (OS) of patients (from the sampling time) according to the proportion of tumor-infiltrating iNKT cells expressing LAG3. Groups were separated using the median percentage of the corresponding marker (*n* = 19 in total, 9–10/group). Comparisons were performed using the log-rank test.

To gain insights into the clinical relevance of the iNKT cell features, we assessed the influence of the iNKT profiles on the PFS and OS of patients with melanoma (**Figure 4**, **Supplementary Table S4**). In blood, high proportions of iNKT cells harboring the EM stage (**Figure 4A**) were linked to longer PFS. In tumor, high proportions of CD86- and NKp46-expressing tumor-infiltrating iNKT cells were associated with better outcomes (**Figure 4B**). Notably, high proportions of LAG3-expressing tumor-infiltrating iNKT cells were highly predictive of shorter OS (**Figure 4C**). Altogether, these observations suggest that iNKT cells are skewed mostly in the tumors of melanoma patients toward a modulated phenotype associated with poorer clinical outcomes, with LAG3 being a critical negative prognostic factor for clinical evolution.

### 3.4 Tumor-infiltrating iNKT cells from melanoma patients harbor a perturbed energetic metabolism—in comparison with circulating iNKT cells from patients or HDs—that affects patient clinical outcomes

**Figure 5:**
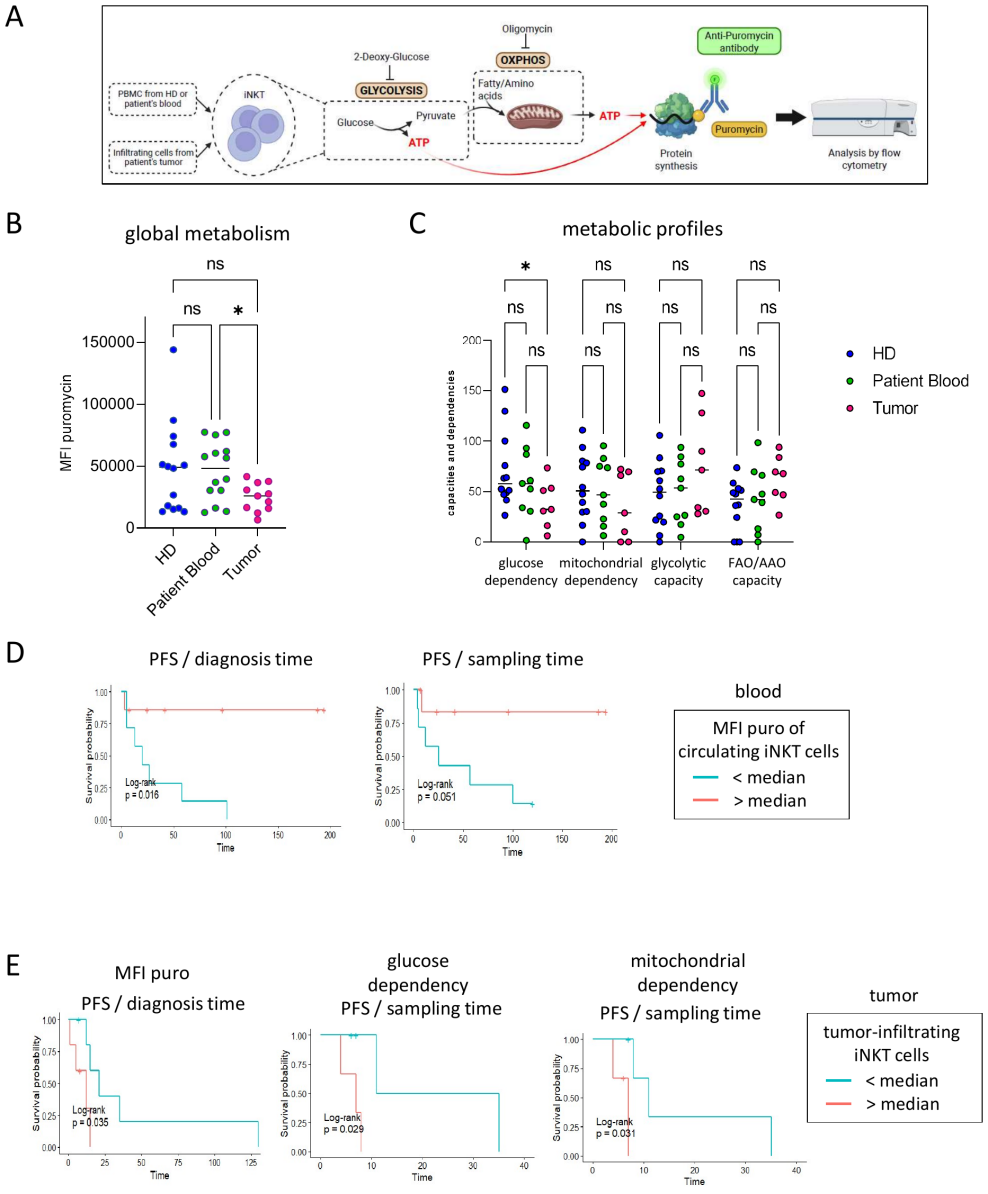
Tumor-infiltrating invariant natural killer T (iNKT) cells exhibit a perturbed metabolism—compared with circulating iNKT cells from patients and healthy donors (HDs)—that influences clinical outcomes. The metabolism of iNKT cells was analyzed using the flow cytometry-based single-cell energetic metabolism by profiling translation inhibition (SCENITH) method. Peripheral blood mononuclear cells (PBMCs; *n* = 14) from HDs and PBMCs (*n* = 14) and tumor-immune infiltrates (*n* = 11) from melanoma patients were cultured in the presence of metabolic inhibitors, and their metabolism was depicted using flow cytometry by measuring the puromycin incorporation intensity. **(A)** Experimental design illustrating the principle of the SCENITH technique. Created using BioRender. **(B)** Comparative global metabolism [mean fluorescence intensity (MFI) of puromycin incorporation] of iNKT cells between the groups (*n* = 14, 14 and 11/group, respectively). *P*-values were evaluated using the non-parametric Kruskal–Wallis test (*solid lines*) or the unpaired Mann–Whitney test (*dotted lines*). **(C)** Comparative metabolic profiles (glycolytic or mitochondrial dependencies and capacities) of the iNKT cells between groups (*n* = 12, 9 and 7/group, respectively). *P*-values were evaluated using the non-parametric two-way repeated measures ANOVA. **(D, E)** Impact of the phenotypic features of iNKT cells on clinical evolution evaluated using Kaplan–Meier analyses. **(D)** Comparative progression-free survival (PFS) of patients [from the diagnosis time (*left panel*) or from the sampling time (*right panel*)] according to the global metabolic level of circulating iNKT cells (*n* = 14 in total, 7/group). **(E)** Comparative PFS of patients from the diagnosis time (*left panel*) or the sampling time (*middle* and *right panels*) according to the level of global metabolism (*n* = 11 in total, 5–6/group), glucose dependency, or mitochondrial dependency (*n* = 7 in total, 3–4/group) of tumor-infiltrating iNKT cells. *P*-values were calculated using log-rank tests. Only significant statistics are displayed on the graphs. ns, non significant.

As metabolism is crucial to dictating cell function, we further investigated whether melanoma could impact the metabolism of iNKT cells. To this end, we used the flow cytometry-based single-cell energetic metabolism by profiling translation inhibition (SCENITH) method, developed by Arguello et al. (34), to determine the metabolic profiles of iNKT cells at a single-cell level (**Figure 5A**, **Supplementary Figures S3A, B**). SCENITH is based on the measurement of the level of protein synthesis (PS) directly within cells by flow cytometry using Puro—a drug able to inhibit translation whose incorporation is a reliable method of measuring the PS level. PS reflects the global metabolic activity of a cell, and changes in PS under the action of metabolic inhibitors allow depicting the metabolic profiles of cells. The incorporation of Puro within iNKT cells was measured in the presence of inhibitors (DG and Oligo) that abrogate the two major metabolic pathways for energy production in cells, i.e., glycolysis and OXPHOS, respectively (**Figure 5A**). These measurements allow assessing the global energetic status [in the absence of inhibitors, e.g., direct mean fluorescence intensity (MFI) of Puro] and calculating the contribution of each pathway to the global metabolism, called dependencies and capacities [see (34, 35)]. Dependencies represent the direct contribution of each pathway to the general metabolism, while capacities depict the ability of the cell to use a pathway when the other is inhibited.

We deciphered the metabolic profiles of the circulating and tumor-infiltrating iNKT cells from patients with melanoma, their link with their phenotypic features, and their impact on patient clinical outcomes in order to determine the intrinsic metabolic features of iNKT cells and the potential perturbations associated with the melanoma context (**Figure 5**). It was found that tumor-infiltrating iNKT cells from patients with melanoma exhibited significantly lower levels of global metabolism compared with the circulating iNKT cells from patients and HDs (**Figure 5B**). This dampened metabolic activity was associated with the metabolic switches within iNKT cells. Indeed, tumor-infiltrating iNKT cells displayed a significantly decreased glucose-dependent metabolism concomitant to a tendency toward increased glycolytic and fatty acid and/or amino acid oxidation (FAO/AAO) capacities compared with the circulating iNKT cells from HDs and/or patients (**Figure 5C**). To further decipher whether the disturbed iNKT cell metabolism affects patient clinical outcomes, we compared the OS and PFS of patients according to the metabolic profiles of their circulating and tumor-infiltrating iNKT cells (**Figures 5D, E**; **Supplementary Figure S3D**, **Supplementary Table S5**). Interestingly, a high level of global metabolism of circulating iNKT cells was associated with a better prognosis (**Figure 5D**), whereas highly metabolic active tumor-infiltrating iNKT cells were associated with shorter PFS (**Figure 5E**). Moreover, in tumors, iNKT cells harboring high glycolytic or mitochondrial dependencies were linked with shorter PFS (**Figure 5E**), whereas iNKT cells exhibiting higher FAO/AAO capacities tended to be associated with better prognosis (**Supplementary Figure S3D**). Overall, these results reveal the significant alterations in the metabolic profiles and the metabolic switches of tumor-infiltrating iNKT cells in patients with melanoma, which influence the clinical outcomes of these patients. This suggests a role of immunometabolism in the subversion of iNKT cells by melanoma.

### 3.5 iNKT cells expressing CD69, PD1, or LAG3 display distinct metabolic activity and profiles than non-expressing cells and switch their metabolic pathways compared with those in HDs

To explore the importance of metabolism on the features of iNKT cells, we simultaneously investigated the metabolic profiles of iNKT cells and the expression of an activation marker (CD69) and ICPs (PD1 and LAG3), whose expression was highly modulated in tumor-infiltrating iNKT cells.

**Figure 6:**
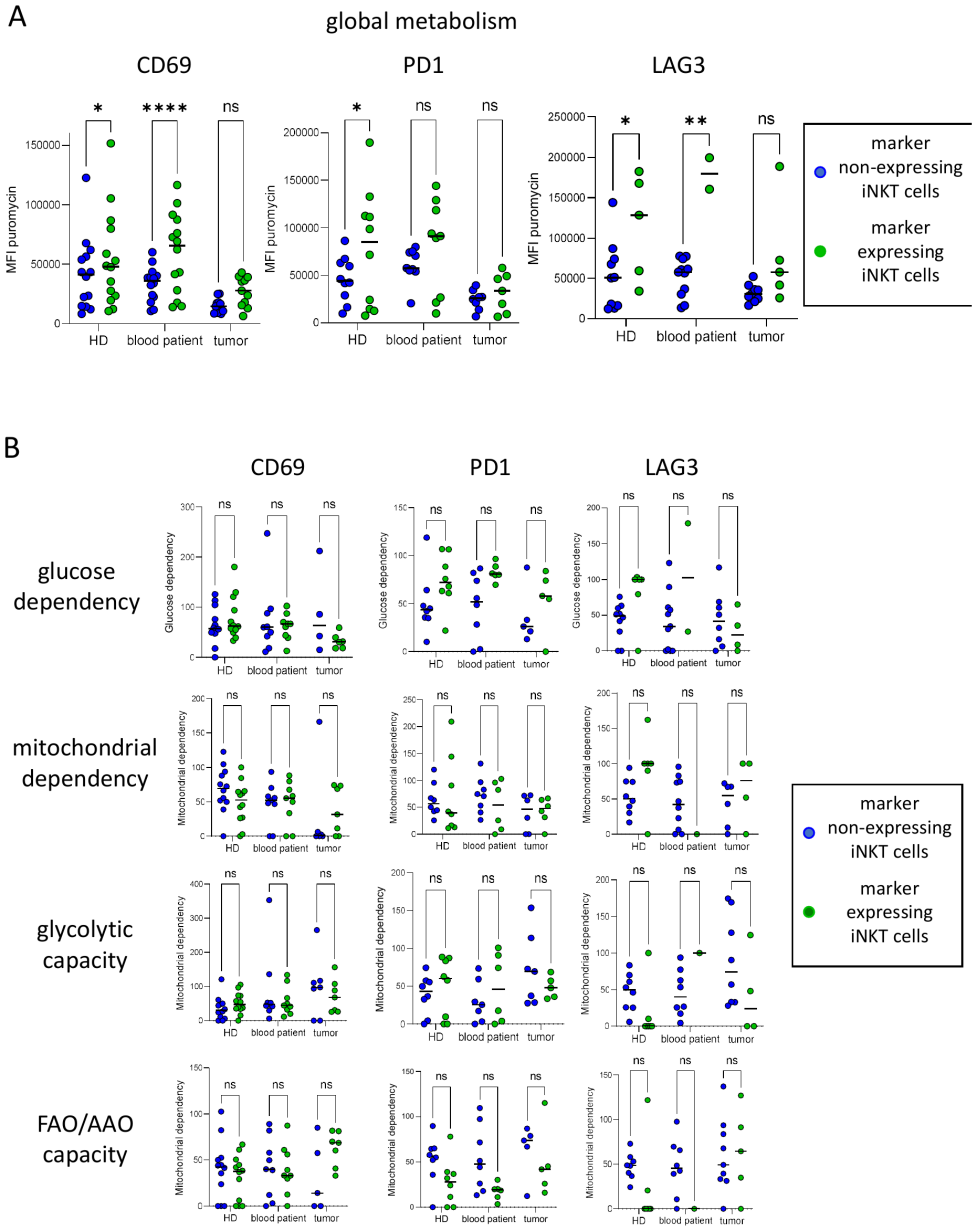
Invariant natural killer T (iNKT) cells expressing CD69, PD1, or LAG3 display distinct metabolic activity and profiles than non-expressing iNKT cells and switch their metabolic pathways compared with healthy donors (HDs). The metabolism of iNKT cells and their immune checkpoint (ICP) profile were simultaneously assessed using the single-cell energetic metabolism by profiling translation inhibition (SCENITH) method combined with ICP profiling. Peripheral blood mononuclear cells (PBMCs) from HDs and PBMCs and tumor immune cell infiltrates from melanoma patients were cultured in the presence of metabolic inhibitors. Intracellular puromycin incorporation (assessed based on the mean fluorescence intensity, MFI) and the surface expression of CD69, PD1, and LAG3 were simultaneously depicted by flow cytometry. **(A)** Comparative levels of global metabolism (MFI puromycin) in iNKT cells expressing CD69 (*n* = 10–14/group), PD1 (*n* = 8–10/group), or LAG3 (*n* = 9–11/group) and the non-expressing iNKT cells in the three groups. **(B)** Comparative proportions of the metabolic dependencies and capacities between CD69- (*n* = 12, 9, and 4–7/group, respectively), PD1- (*n* = 8–9, 6–8, and 5–7/group, respectively), or LAG3-expressing (*n* = 6–10, 1–11, and 4–8/group, respectively) and non-expressing iNKT cells in the three groups. *P*-values were calculated using two-way repeated measures ANOVA with Sidak’s multiple comparison posttest. Only significant statistics are displayed on the graphs. *p < 0.05, **p < 0.01, ****p < 0.0001; ns, non significant.

Interestingly, we observed distinct metabolic profiles of iNKT cells according to their activation status (expression of CD69) and ICP profile (expression of PD1 and LAG3) (**Figure 6**). The expression of CD69, PD1, or LAG3 by iNKT cells from the blood of HDs and patients was associated with a higher level of global metabolism compared with non-expressing cells, whereas the metabolic activity did not differ between the CD69-, PD1-, or LAG3-expressing and non-expressing tumor-infiltrating iNKT cells (**Figure 6A**). In addition, while the CD69-expressing and non-expressing iNKT cells displayed similar metabolic profiles in the three groups (**Figure 6B**, left panels), despite being non-significant, the PD1-expressing iNKT cells slightly tended to exhibit a higher glucose dependency and a lower FAO/AAO capacity compared with the PD1-negative iNKT cells (**Figure 6B**, middle panels). LAG3 expression by the iNKT cells from HDs tended to be linked with higher glucose and mitochondrial dependencies and concomitant lower capacities, whereas in patients with melanoma, the LAG3-expressing iNKT cells displayed similar dependencies to non-expressing cells, demonstrating the metabolic flexibility of LAG3^+^ iNKT cells in the melanoma microenvironment. These observations may underscore that iNKT cells display distinct metabolic profiles depending on their activation status and ICP profile, underlining critical connections between the features and metabolic patterns of iNKT cells.

## 4 Discussion

**Figure 7:**
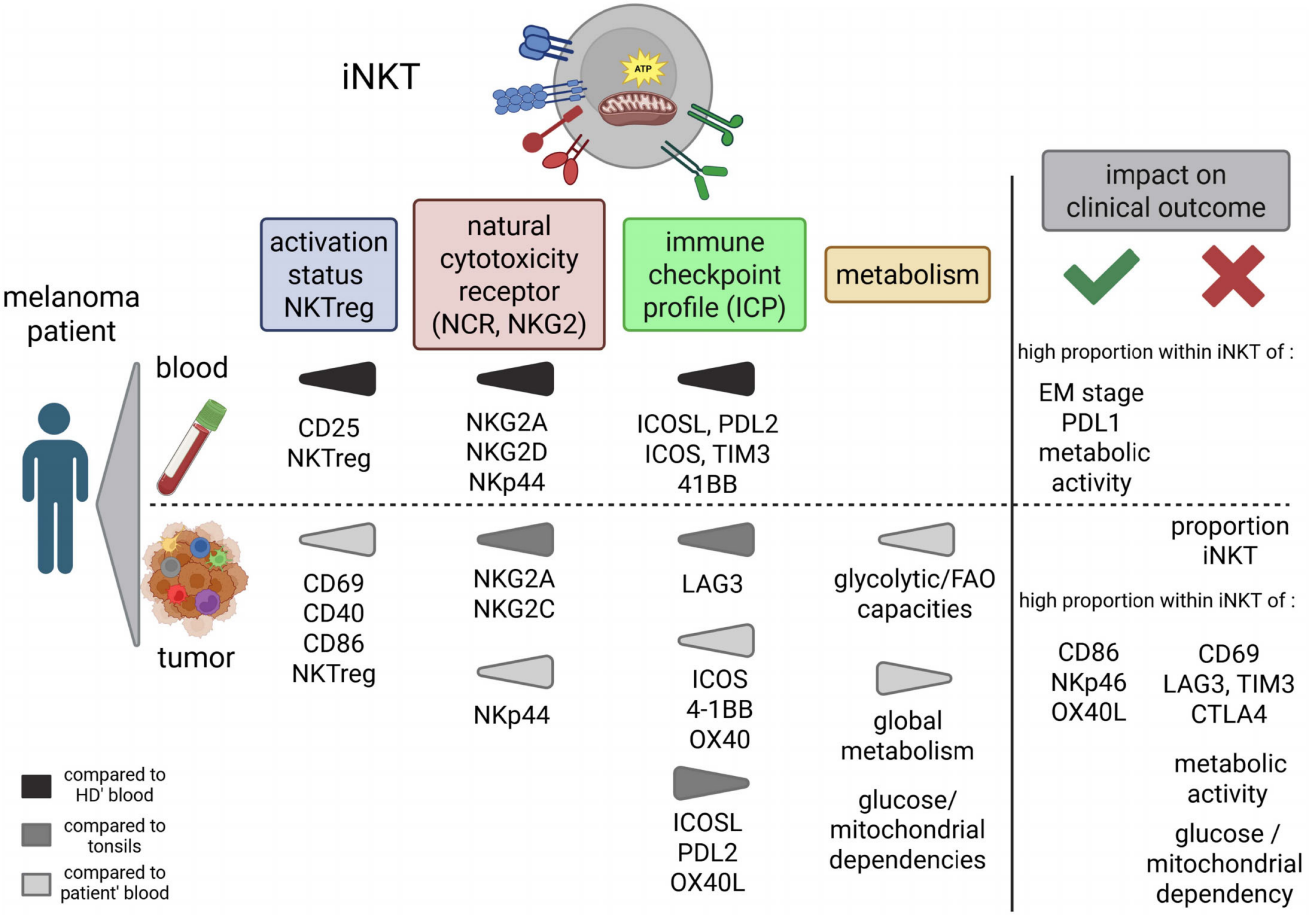
Graphical summary of the main perturbations of circulating and tumor-infiltrating invariant natural killer T (iNKT) cells in melanoma patients and their impact on clinical outcomes. The phenotypic and metabolic features of circulating and tumor-infiltrating iNKT cells from melanoma patients were explored by flow cytometry. The impact of the features of iNKT cells on patients’ clinical evolution was further evaluated. *Left part*: Perturbations observed in both the blood and tumor of melanoma patients compared with the corresponding control groups. *Right part*: Highlighting iNKT cell-based prognostic factors for clinical evolution. The circulating iNKT cells from melanoma patients exhibited a modulated activation status (increased proportion of CD25^+^ cells) and increased iNKTreg profile, a perturbed natural cytotoxicity receptor (NCR) and NKG2 profile (increased proportion of NKG2A^+^, NKG2D^+^, and NKp44^+^ cells), and a deregulated immune checkpoint (ICP) profile (increased proportions of ICOSL^+^, PDL2^+^, ICOS^+^, TIM3^+^, and 41BB^+^ cells) compared with the iNKT cells from healthy donors (HDs). Their metabolism remained similar to that in HDs. The tumor-infiltrating iNKT cells from melanoma patients exhibited a modulated activation status, an increased iNKTreg profile, and a perturbed NCR/NKG2 profile (increased proportions of NKG2A^+^ and NKG2C^+^ cells), as well as a deregulated ICP profile (increased LAG3^+^, ICOS^+^, 4-1BB^+^, and OX40^+^ cells and decrease in ICOSL^+^, PDL2^+^, and OX40L^+^ cells) and a skewed metabolism [increased glycolytic/fatty acid oxidation (FAO) capacities and decreased global metabolism level and glucose/mitochondrial dependencies] compared with HDs. High proportions of circulating iNKT cells from melanoma patients in the effector memory (EM) stage, expressing PD-L1 or exhibiting a high level of metabolic activity, are associated with good prognosis. High proportions of tumor-infiltrating iNKT cells, or high proportions of tumor-infiltrating iNKT cells expressing CD69, NKG2C, LAG3, CTLA4, or TIM3 or exhibiting a high level of metabolic activity or high glucose/mitochondrial dependencies, are associated with worse prognosis. In contrast, high proportions of tumor-infiltrating iNKT cells expressing CD86, NKp46, and OX40L or exhibiting a high FAO/amino acid oxidation (AAO) capacity are rather associated with a good prognosis. This figure was created using the BioRender scientific illustration tool.

iNKT cells are fascinating cells due to their unique properties and functional plasticity, which render them very attractive for immunotherapy. iNKT cells exert both effector and regulatory functions, allowing them to orchestrate immune responses. However, these rare but potent antitumor effectors have not been extensively explored in melanoma, in particular within the TME. Previous investigations were limited to the identification of iNKT cells within primary tumors and reports of alterations in the frequencies and functions of circulating iNKT cells mostly in the colon, in prostate cancer, and in neuroblastoma (15). There is little information available on their features and clinical significance, as well as their ICP expression and metabolic fitness in the context of cancer. We provided here a detailed investigation of circulating and tumor-infiltrating iNKT cells in terms of their frequency, phenotype, properties, and relationship with clinical evolution in patients with melanoma. Our study highlights that the features of iNKT cells make them promising potential biomarkers of the clinical evolution in melanoma and provide a better understanding of the physiopathology and hijacking of iNKT cells in melanoma (see Graphical abstract, **Figure 7**). This may help in the design of new therapeutic approaches that exploit the potential of iNKT cells to improve patient outcomes.

We highlighted similar proportions of circulating iNKT cells between melanoma patients and HDs, but an increased frequency of iNKT cells in tumors compared with control non-tumoral tissues, revealing an active migration of iNKT cells toward melanoma. The recruitment of iNKT was higher in cutaneous tumors compared with lymph node metastasis, suggesting favorable signals in the skin that attract iNKT cells. Interestingly, despite similar circulating iNKT frequencies in men and women, a higher infiltration of tumors by iNKT cells was observed in women compared to men, highlighting a gender-dependent recruitment of iNKT cells to tumors. In a large cohort of HDs, a gender bias in the frequency of circulating iNKT cells has been observed, with higher iNKT cell proportions in women compared to men (36). There is increasing evidence that the male and female innate and adaptive immune responses against tumors are not equivalent (37). Sex-based differences in immunity have been observed especially for CD8^+^ T cells and NK cells, and this may impact the antitumor responses, drive disparate tumor control, and influence therapeutic outcomes. Our observations suggest that the behavior of iNKT cells may also be gender-biased in cancer.

For the first time, we depicted the extensive phenotypic, metabolic, and functional features of iNKT cells in patients with melanoma, both in blood circulation and within the TME. We investigated iNKTreg cells and the expression of a large panel of NCR, NKG2, and ICP/ICP-L by iNKT cells in the context of melanoma and highlighted crucial clinical correlations. Such exploration is important as a modulated NCR/NKG2 expression profile may affect the capacity of iNKT cells to recognize and kill target tumor cells. Moreover, assessment of the ICP profile informs on the capacity of iNKT cells to be targeted by the ICP blockers currently used in patients with melanoma.

The circulating and tumor-infiltrating iNKT cells from melanoma patients showed elevated proportions of iNKTreg cells and exhibited higher levels of activation markers, NKR/NKG2 (such as NKG2A, NKG2C, and NKG2D), and ICP (particularly ICOS, TIM3, and/or LAG3) compared with the control groups. This is consistent with observations on a B16 melanoma mouse model in which iNKT cells displayed dysfunctional states with increased expression of the exhaustion markers (PD1, CTLA4, and TIM3) and of NKG2D, as well as a low level of IFNγ production associated with IL-4, IL-10, and IL-17 production in the TME (23). We identified modulations on iNKT cells both in the blood and tumor as critical negative prognostic factors for clinical evolution. A high proportion of tumor-infiltrating iNKT cells was associated with worse clinical outcomes. We also clearly identified that high proportions of LAG3^+^, TIM3^+^, and CTLA4^+^ tumor-infiltrating iNKT cells were predictive of poor clinical outcomes. Such correlations have never been reported to date for iNKT cells in patients with cancer.

iNKT cells are known to exert antitumor activity. However, our work demonstrates that melanoma may hijack iNKT cells to exhibit tumor-promoting functions. Indeed, we observed an accumulation of FoxP3^+^ iNKTreg cells within melanoma tumors, a disturbed NCR/NKG2 profile, and a skewed ICP expression toward LAG3, CTLA4, and TIM3, which were associated with early relapse and shorter survival. Melanoma may favor the differentiation of iNKT cells toward an exhausted phenotype by chronically stimulating them with lipids that accumulate in tumor cells. This could be a mechanism of escape from iNKT cell antitumor function. It has also been demonstrated that soluble factors secreted by tumor cells could drive such skewing and impair iNKT cell function. Indeed, iNKT cells expanded from PBMCs and co-cultured with melanoma tumor cells exhibited a dampened NKG2D expression and reduced cytotoxic capacities in an IDO-1- and PGE2-dependent manner (38). Such an impaired cytotoxic function and the modulation of the immunoregulatory profile have also been described for other antitumor effectors, in particular NK and CD8 T cells, in the blood of melanoma patients compared with HDs and in metastatic regional lymph nodes compared with non-metastatic lymph nodes (39, 40). Such dysfunctions on multiple key antitumor effectors may sustain melanoma-associated immunosuppression and disease progression.

Our work revealed that LAG3, TIM3, and CTLA4 are crucial ICPs on iNKT cells favoring immune escape and tumor progression. These observations thus pointed out that iNKT cells may be interesting cells to study in the setting of current immunotherapies with ICP blockers as they display a perturbed ICP panel expression and could be targeted by these therapies. Our data also validated a combinatorial therapeutic approach since the three major inhibitory ICPs that currently undergo clinical developments are expressed by tumor-infiltrating iNKT cells. Our data suggest that anti-LAG3 antibodies may be worth examining to reinvigorate dysfunctional tumor-infiltrating iNKT cells. However, many steps remain to be solved and investigated in order to fully exploit LAG3 as a novel immunotherapeutic target. Interestingly, we previously characterized iNKT cells in the context of ICP blockers in patients with melanoma and highlighted the crucial influence of the features of iNKT cells on the ability of patients to respond to anti-program cell death protein 1 (PD-1) therapy (41). Indeed, we observed lower frequencies of IFNγ/TNFα-secreting iNKT cells in response to phorbol myristate acetate (PMA)/ionomycin stimulation in responder patients compared with non-responder patients before the start of anti-PD-1 treatment. Moreover, we detected decreased frequencies of CD40^+^ and GITR^+^ iNKT cells and increased frequencies of TIGIT^+^ iNKT cells in responders compared with non-responder patients during the course of treatment. Thus, the phenotypic profiles and functional capacities of iNKT cells not only dictate the clinical outcomes but are also critical in the immunotherapeutic response.

Immunometabolism is of growing interest and is considered as an interesting therapeutic target to orientate and fine-tune immune responses. As metabolism crucially regulates the immune cell function, their metabolic reprogramming could be a pathway of immune subversion and tumor immune escape. However, currently, the metabolic features and perturbations of immune cells in melanoma, together with their functional impacts, are unknown. Our work, for the first time, revealed significant alterations and major disturbances in the metabolic activity and potential metabolic switches of tumor-infiltrating iNKT cells in patients with melanoma. This suggests a role for immunometabolism in the subversion of iNKT cells by melanoma. The decreased dependencies concomitant to tendencies to increased capacities reflect the high metabolic flexibility of iNKT cells in the TME. The dampened glucose dependency observed in tumor-infiltrating iNKT cells is in line with observations in tumor-bearing mice, where circulating and tumor-infiltrating iNKT cells exhibited an unbalanced metabolism characterized by a suppressed glucose metabolism (decreased expression of the transporters Glut1 and Glut3 and of the glycolysis-related enzymes) (23).

We not only demonstrated that melanoma severely impaired the phenotypic and metabolic features of iNKT cells but also unveiled the critical interplay between the metabolic pathways and the iNKT cell features. We underlined that iNKT cells display distinct metabolic profiles depending on their activation status and ICP profile, supporting critical connections between the iNKT cell features and metabolic patterns. Moreover, the metabolism of circulating and tumor-infiltrating iNKT cells was pivotal for clinical outcomes in melanoma. Thus, tumors, through a metabolic hostile microenvironment, could manipulate the metabolism of iNKT cells to hijack their function and escape immune surveillance. Interestingly, previous studies highlighted a link between immune cell function and metabolism, as a depression in NK cell activity in patients with cancer correlated with an increase in lactate dehydrogenase (LDH) release and loss of the components of the secretory killing pathway (42).

How tumors reprogram iNKT cell metabolism in patients with cancer is currently unknown. A study highlighted that lactic acid from the TME reduced the PPARγ expression in intratumoral iNKT cells and subsequently dampened the cholesterol synthesis required for IFNγ production (43). Thus, an impaired lipid biosynthesis may hinder the antitumor efficacy of tumor-infiltrating iNKT cells. The combination of PPAR agonists with αGalCer enhanced the iNKT cell-mediated antitumor responses and survival of tumor-bearing mice (43). Other studies revealed that metabolism controls the molecular processes of NKT cells to respond to lipid antigens and subsequent functions (32, 33). Indeed, glycolysis is required for the antigen presentation and effector functions of iNKT cells during viral infection (32). The synthesis and the processing of glycolipids are dependent on key metabolic processes and may steer iNKT cells toward adopting a Th1 or a Th2 signature (33). In addition, in a tumor-bearing mouse model, the genes of the key enzymes involved in cholesterol synthesis were downregulated in NKT cells from the blood and tumor (23).

Our study highlights that metabolic reprogramming could trigger iNKT cell subversion in melanoma and open the way for metabolic interventions to rescue iNKT cells from an altered metabolic reprogramming and restore their functions and antitumor responses. To connect the metabolic profiles with the surface checkpoint/activation markers, we simultaneously investigated the metabolic profiles of iNKT cells and the expression of an activation marker (CD69) and ICPs (PD1 and LAG3), whose expression was highly modulated in tumor-infiltrating iNKT cells, within the same cells. This allows directly evaluating the expression levels of the checkpoint/activation markers on iNKT cells with a specific metabolic profile and the metabolic features of iNKT cells harboring or not specific checkpoint/activation markers. Tumor-infiltrating iNKT cells, being the most glycolytic, are rather PD1-negative and LAG3-negative, suggesting that subverted PD1^+^ and/or LAG3^+^ iNKT cells exhibit a rather low glycolytic capacity, but a high mitochondrial dependency. In patient blood and tumor, CD69-expressing iNKT cells displayed a higher global metabolism than non-expressing cells, which was associated with a longer PFS in blood, but shorter PFS for tumor. Metabolic skewing of the iNKT cells in melanoma is characterized by rather low metabolic dependencies and high metabolic capacities. In patient blood, the expression of CD69 did not discriminate cells with these distinct dependencies/capacities. In patient tumor, CD69-expressing cells exhibited a rather low glucose dependency, a parameter associated with longer PFS, hence better prognosis, and a high FAO/AAO capacity, whereas it was the opposite for PD1-expressing cells. The link between metabolism and immune function supports that targeting metabolism emerges as a potent means to regulate iNKT cells and control the immune response. The PI3K/AKT and mTOR pathways are responsible for glycolysis induction, which are counterbalanced by the AMPK signaling pathway that drives OXPHOS (44). Reversion of metabolic disturbances can be achieved by acting on these signaling pathways, but also on the availability of nutrients (nutrient deprivation/overdose) or by regulating nutrient transporters, as already demonstrated for DCs (35). Further understanding of the relationship between metabolic fate and phenotypic/functional defects in the tumor context will provide information for the development of novel strategies manipulating iNKT cell metabolism for cancer therapy.

Our study reveals major phenotypic and metabolic disturbances in circulating and tumor-infiltrating iNKT cells in melanoma with clinical impacts. Such phenotypic and metabolic alterations in iNKT cells driven by melanoma may contribute to an inefficient antitumor immune response by directly impairing the killing of tumor cells and by altering the subsequent interactions of iNKT cells with other immune effectors. Melanoma disables iNKT cells by altering their antitumor potentialities, by driving metabolic switches, or by skewing their ICP expression. Our findings uniquely provide insights into the mechanisms of iNKT subversion in melanoma and pave the way for innovative therapeutic options exploiting metabolic pathways and/or disturbed ICP profiles to overcome immune subversion and restore immune function, ultimately improving the success of immunotherapies.

Numerous studies in preclinical mouse models and clinical trials of patients with cancer have proven that the restoration of iNKT cell function may stimulate potent antitumor immune responses (9, 16), proving that exploiting iNKT cells in cancer immunotherapy is safe and could favor therapeutic benefits. iNKT cell-based strategies could serve as a potent adjuvant for cancer immunotherapy (9, 12, 45). iNKT cell agonists, such as αGalCer and optimized analogues, enhance the adjuvant effects of iNKT cells and trigger strong Th1 responses. These agonists have entered clinical trials as vaccine adjuvants, in particular in melanoma. Targeting of the tumor antigen together with αGalCer to cDC1s *via* a Clec9α-coated nanoparticle vaccine strongly induced the expansion of antigen-specific CD8 T cells *ex vivo* from melanoma patients (46) and promoted the antitumor responses *in vivo* in humanized mice (47). The use of iNKT cell agonists in combination with ICP blockers is also promising. iNKT cells, as conventional T cells, upregulate PD-1 upon activation, and as shown in our study, tumor-infiltrating iNKT cells in patients with melanoma express PD-1, thus being sensitive to ICP blockers. Interestingly, the blockade of PD1 simultaneously with αGalCer resulted in a persistent antitumor immune response in a B16 melanoma model (48). Such combination therapy demonstrated efficacy in melanoma mouse models with anti-PD-1 (18) and anti-CTLA4 (49), even allowing overcoming resistance to anti-PD-1 (50). Our data showed that LAG3 is highly upregulated in tumor-infiltrating iNKT cells and is associated with worse clinical outcomes, sustaining the targeting of LAG3 together with iNKT cell agonists.

iNKT cells have attracted considerable interest in cancer therapy; however, iNKT cell-based immunotherapies have yielded inconsistent outcomes and indistinct clinical responses. There is an unmet need to pursue combination approaches targeting iNKT cells to better harness their antitumor potential in the clinic. By unveiling new key features and skewing details of the iNKT cells in melanoma, our study opens an opportunity to better harness the potential of iNKT cells for cancer immunotherapy and to develop new combination strategies targeting ICP or cell metabolism.

Despite major advances, our study suffers from some limitations that need to be acknowledged. Firstly, we used tonsils as the control for tumor tissue (as the majority of samples were derived from metastatic lymph nodes); however, tonsils may not be the best control. Indeed, tonsils and lymph nodes both contain abundant iNKT cells. However, their proportions, activation states, and functional profiles are very different, implying that comparisons of metastatic lymph nodes with tonsils should be taken with caution. Secondly, for some assessments, especially when evaluating the link between ICP expression by iNKT cells and their metabolic profile, very few samples were analyzed. We actually attempted to exploit maximal information out of the data. Although the conclusions need to be interpreted with caution, this study provides unique insights into the pathophysiology of iNKT cells in melanoma, which requires further validation in bigger cohorts.

Altogether, our study demonstrates that melanoma hijacks iNKT cells and reprogram their energetic metabolism to escape from immune control. Further insights into the biology of iNKT cells in relation to disease outcomes within the TME is critical for harnessing iNKT cells for cancer immunotherapy and the development of effective antitumor therapies. Understanding the functional plasticity and metabolic regulation of iNKT cell function and dysregulation in cancer would allow a better understanding of the immune subversion by tumors and paves the way for the appropriate tuning of metabolic pathways to rescue iNKT cells from tumor hijacking in order to restore their potentialities to trigger antitumor responses and enhance the efficacy of current immunotherapies.

## Data Availability

The original contributions presented in the study are included in the article/**Supplementary Material**. Further inquiries can be directed to the corresponding author.
