# Supplementary material for: Melanoma causes phenotypic modulations and metabolic switches of iNKT cells influencing clinical outcomes
Source: Front Immunol. 2026 Jan 5;16:1703754. doi: 10.3389/fimmu.2025.1703754 (PMC12813057; doi:10.3389/fimmu.2025.1703754)

Supplementary Material

1. Supplementary Figure legends

Supplementary Figure 1: FACS gating strategy to depict iNKT cells and their phenotypic and metabolic features

PBMC and tonsil-derived cell suspensions from HD as well as PBMC and tumor-immune cell infiltrates from melanoma patients were submitted to flow cytometry analysis to depict the phenotypic and metabolic features of iNKT cells. A/ Cell debris and cell doublets were then excluded using the FSC and SSC parameters. Viable (Live&Dead^{-}) CD45^{+} cells were gated prior to iNKT cell identification as CD45^{+} CD3^{+} iNKT^{+} cells. Representative dot plots of one sample per group. B-F/ Representative dot plots illustrating expression of activation molecules (CD40 and CD69) (B), of CD56 and Treg-associated markers (FoxP3 and CD25) (C), of NCR NKp30, NKG2A, NKG2D (D), and of immune checkpoints (PD1, OX40, ICOS) by iNKT cells (E), and their stage of differentiation (F). Dot plots pre-gated on alive CD45^{+} CD3^{+} iNKT cells. One representative dot plot for HD and for patient tumor sample. Control isotypes were used to set the threshold of positivity for each marker.

Supplementary Figure 2: Phenotypic profiling of circulating and tumor-infiltrating iNKT cells in melanoma patients compared to healthy samples

PBMC (n=14) and tonsil-derived cell suspensions (n=9) from HD as well as PBMC (n=19-24) and tumor-immune infiltrates (n=17-24) from melanoma patients were submitted to flow cytometry analysis to depict their activation status, KLR and NCR expression, ICP and ICP-L profile, and regulatory status. A-D/ Comparative proportions of iNKT expressing the activation marker CD86 (A), the KLR/NCR NKG2C, NKp30, NKp46 (B), the ICP-ligands OX40L, PDL1, 4-1BBL (C), and the ICP CTLA4, 4-1BB, OX40. E/ The differentiation stage of iKNT cells was evaluated by flow cytometry using CD45RA and CD27 markers: naïve (CD45RA^{+} CD27^{+}), CM (CD45RA^{-} CD27^{+}), EM (CD45RA^{-} CD27^{-}) and

EMRA (CD45RA$^+$ CD27$^-$). F/ Comparative proportions of Treg iNKT cells based on combined FoxP3 and CD25 expression. P-values calculated using the non-parametric unpaired Mann-Whitney test. Only significant statistics are displayed on graphs.

Supplementary Figure 3: Metabolic features of iNKT cells and link with clinical outcome

The metabolism of iNKT cells was analyzed using the flow cytometry-based SCENITH method. PBMC and tonsil-derived cell suspensions from HD as well as PBMC and tumor-immune infiltrates from melanoma patients were cultured in the presence of metabolic inhibitors and their metabolism was depicted by flow cytometry by measuring puromycin incorporation intensity. The impact of the phenotypic features of iNKT cells on clinical evolution was evaluated using Kaplan-Meier analyses. A/ The metabolic features of iNKT cells were assessed using the SCENITH technique. Histogram depicting the incorporation of puromycin by iNKT in the presence of the inhibitors of glycolysis (2-Deoxy-Glucose, DG) or OXPHOS (Oligomycin, O) metabolic pathways or both inhibitors (DGO). MFI of puromycin is further used to calculate glycolytic and mitochondrial dependencies and capacities. B/ Graphs displaying the intensity of puromycin incorporation (MFI) in the presence of 2-Deoxy-Glucose (DG), Oligomycin (O) or both (DGO) in order to subsequently calculate the glycolytic and mitochondrial dependencies/capacities for iNKT cells from HD (n=12), patient blood (n=9) and patient tumor (n=7). The central line indicates median, the box contains the 25th to 75th percentile, and whiskers the min and max values. C/ Heat maps illustrating the level of global metabolism (left panel) and the metabolic dependencies/capacities (right panel) of iNKT cells from HD blood, patient blood and patient tumor, at basal state. D/ Comparative PFS of patients (from sampling time, left panel) or from diagnosis time, right panel) according to the level of FAO capacity of tumor-infiltrating iNKT cells (n=7 in total, 3-4/group).

2. Supplementary Tables

Supplementary Table 1: Clinical features of patients from whom blood was used

									from diagnosis time		from sampling time	
patient #	type	gender	age (years)	breslow (mm)	clark	ulceration	treatment before sampling	stage at sampling time	PFS (months)	OS (months)	PFS (months)	OS (months)
1	PBMC	F	49	0.3	II	no	surgery, chemotherapy	IA (T1aN0M0)	59	70	6	16
2	PBMC	M	80	13	IV	yes	-	IIC (T4bN0M0)	>70	>70	>42	>42
3	PBMC	M	67	0.96	III	no	-	IIIA (T1aN1M0)	48	>250	>42	>42
4	PBMC	M	58	1.1	III	no	-	IB (T2aN0M0)	>69	>69	>42	>42
5	PBMC	M	58	4	V	yes	surgery	IIC (T4bN0M0)	58	62	6	9
6	PBMC	M	57	14	V	no	sugery	IV (T4aN3M1c)	13	>52	1	>40
7	PBMC	M	43	1.5	III	no	surgery	IIIA (T2aN1aM0)	>70	>70	>40	>40
8	PBMC	F	80	unknown primitive			-	III or IV				44
9	PBMC	M	32	1.4	IV	no	-	IIIA (T2aN2aM0)	>120	>120	>120	>120
10	PBMC	M	45	1.3	III	no	-	IIIA (T2aN2aM0)	>30	>30	>30	>30
11	PBMC	M	73	2.6	IV	yes	-	IIB (T3bN0M0)		132		132
12	PBMC	F	70	3	III	yes	-	IIB (T3bN0M0)	>97	>97	>97	>97
13	PBMC	M	54	1	III	yes	surgery	IV (T1bN1bM1a)	228	275	0	45
14	PBMC	F	26	1.02	III	no	-	IB (T2aN0M0)	>76	>76	>76	>76
15	PBMC	M	72	1.399	IV	no	-	IB (T2aN0M0)		104		104
16	PBMC	F	52	1.2	III	no	chemotherapy	IIIA (T2aN2M0)	44	142	6	73
17	PBMC	F	79	unknown primitive			-					
18	PBMC	F	20	1.34	III	no	-	IB (T2aN0M0)	>148	>148	>148	>148
19	PBMC	F	51	0.8	III	no	-	IA (T1aN0M0)	51	>74	24	
20	PBMC	F	64	1.2	IV	no	-	IB (T2AaN0M0)	>148	>148	>146	>146
21	PBMC	F	77	2.28	IV	yes	-	IIA (T2bN0M0)	>147	>147	>142	>142
22	PBMC	M	62	0.85	IV	no	-	IA (T1aN0M0)	>151	>151	>151	>151
23	PBMC	M	79	2.8	IV	yes	-	IIB (T3bN0M0)	>45	157	>45	157
24	PBMC	M	62	2.1 / 0.48	IV / II	nd	surgery, radiotherapy	IV	51	77		22
25	PBMC	M	59	5	IV	yes	-	IV (T4bN1aM1c)	27	64	0	26
26	PBMC	F	49	1.01	III	no	-	IB (T2aN0M0)	>161	>161	>161	>161
27	PBMC	F	53	1.21	IV	no	-	IB (T2aN0M0)	>72	>72	>71	>71
28	PBMC	M	59	3	III	no	-	IIA (T3aN0M0)	>74	>74	>73	>73
29	PBMC	M	23	1.8	III	no	-	IB (T2aN0M0)	66	70	66	70
30	PBMC	M	69	2.25	IV	yes	-	IIB (T3bN0M0)	>71	94	>71	94
31	PBMC	M	60	3.2	III	yes	-	IIB (T3bN0M0)	>67	>67	>66	>66
32	PBMC	M	76	2.2	IV	no	-	IIA (T3aN0M0)	>74	>74	>74	>74
33	PBMC	F	75	1.05	IV	no	deticene (5y before sampling), vemurafenib (2y before sampling)	IV (T2aN3M1b)	60	>180	12	>36
34	PBMC	F	63	1.68	III	yes	-	IIA (T2bN0M0)	>24	>24	>24	>24
35	PBMC	M	31	1.66	IV	no	surgery	IIIb (T2aN2bM0)	5	>186	>120	>182
36	PBMC	M	73	1.3	?	?	no	IB (T2aN0M0)	101	>102	100	>101
37	PBMC	F	65	1.3	III	no	no	IB (T2aN0M0)	>188	>188	>187	>187
38	PBMC	F	74	5	IV	yes	no	IIID (T4bN3cM0)	5	21	5	21
39	PBMC	M		1.58	III	yes	no	IIIB (T2bN1bM0)	13	106	12	105
40	PBMC	M	62	3.4	IV	no	no	IIA (T3aN0M0)	58	75	57	74
41	PBMC	M	52	?	?	?	surgery	IV	20	90	4	4
42	PBMC	F	69	1.2	IV	no	no	IB (T2aN0M0)	27	>123	25	>121
43	PBMC	F	43	0.9	III	no	no	IA (T1bN0M0)	>25	>25	>24	>24
44	PBMC	M	75	8	IV	yes	surgery	IIID (T4bN3M0)	3	19	8	18
45	PBMC	M	70	1.3	III	no	no	IB (T2aN0M0)	>97	>97	>96	>96
46	PBMC	F	48	1.2	IV	no	no	IB (T2aN0M0)	>194	>194	>194	>194
47	PBMC	F	83	2.25	IV	yes	no	IIB (T3bN0M0)	>42	120	>42	120
48	PBMC	F	46	3.96	IV	yes	no	IIIC (T3bN1aM0)	>8	>8	>7	>7

Supplementary Table 2: Clinical features of patients from whom tumors were used

									from diagnosis time		from sampling time	
patient #	type	gender	age (years)	breslow (mm)	clark	ulceration	treatment before sampling	stage at sampling time	PFS (months)	OS (months)	PFS (months)	OS (months)
49	cutaneous metastasis	M	65	1.5	IV	no	surgery	IV (T2bNxM1c)	86	134	11	50
50	lymph node metastasis	M	78	2	IV	yes	surgery	IV (T2aN0M1c)	9	10	0	1
51	lymph node metastasis	M	53	3.5	IV	no	surgery, interferon	IV (T3aN3M1a)	58	>158	2	>35
52	lymph node metastasis	M	61	1.7	IV	yes	surgery	IV (T2bN1bM1c)	109	141		31
53	cutaneous metastasis	M	90	6	IV	yes	surgery	IV (T4bN0M1)	0		0	
54	hepatic metastasis	F	49	0.3	III	no	surgery	IV (T1aN0M1c)	58	69	4	15
55	lymph node metastasis	F	31	2	3	no	surgery	IV (T2aN3M1a)	14	30	1	16
56	lymph node metastasis	F	82	2.8	IV	yes	surgery	IV (T3bN2bM1a)	18	>60	>34	>34
57	lymph node metastasis	M	46	6.25	IV	yes	no	IV (T4bN1bM1c)	7	>182	>174	>174
58	lymph node metastasis	F	44	11	V	yes	no	IIIC (T4aN2bM0)	7	>134	6	>133
59	cutaneous metastasis	F	76	5	IV	yes	chemotherapy	IV (T4bN2bM1c)	6	21	0	3
60	lymph node metastasis	M	70	7	IV	yes	no	IV (T4bN1bM1c)	9	>141	0	>125
61	primary tumor	M	39	9	IV	yes	no	IIIC (T4bN1M0)	22	57	22	57
62	lymph node metastasis	F	96	unknown primitive			no	IIIB (TxN1bM0)				
63	lymph node metastasis	M	73	3	III	no	no	IIIB (T3aN1aM0)	218	253	27	37
64	lymph node metastasis	M	25	unknown primitive			no	IIIB (TxN1bM0)	0	30	0	30
65	lymph node metastasis	M	47	unknown primitive			no	IIIC (TxN3M0)	15	20	0	5
66	lymph node metastasis	M	50	1.5	III	no	no	IIIA (T2aN1aM0)	3	30	17	27
67	lymph node metastasis	F	59	2.5	IV	nd	interferon (4y before sampling)	III	70	97	12	26
68	lymph node metastasis	F	72	3.09	IV	yes	surgery	IIIC (T3bN3M0)	2	6	0	3
69	lymph node metastasis	M	46	1.4	III	yes	surgery	IIIC	0	57	0	57
70	lymph node metastasis	M	79	1.58	III	yes	surgery	III	13	106	12	105
71	lymph node metastasis	F	70	0.78	III	nd	chemotherapy, DC vaccine (1y before sampling)	III	36	107	0	5
72	lymph node metastasis	M	31	1.66	IV	nd	surgery	III	>159	>159	>157	>157
73	lymph node metastasis	F	50	2.2	III	yes	no	IIIC (T3bN3M0)	16	49	17	32
74	lymph node metastasis	F	35	2.75	IV	yes	no	IIIC (T3bN1bM0)	1	70	0	69
75	lymph node metastasis	F	59	1.15	III	nd	no	IIIB (T2N1bM0)	35	39	0	4
76	lymph node metastasis	F	39	1.1	IV	no	no	III				
77	cutaneous metastasis	M	27	2.64	IV	nd	surgery, radiotherapy, interferon (3y before sampling)	IV	15	84		22
78	lymph node metastasis	F	56	12	V	no	no	IIIA (T4aN1aM0)	>20	>20	>18	>18
79	lymph node metastasis	F	40	unknown primitive			no	IV	3	3	2	2
80	lymph node metastasis	M	51	4	IV	no	no	IIIC (T4N3M0)	17	28	14	25
81	lymph node metastasis	M	86	10	IV	no	surgery	IV	6	22	0	10
82	lymph node metastasis	M	23	2.64	IV	no	no	IIIC (T3N3M0)	13	83	0	69
83	lymph node metastasis	F	65	1.2	IV	no	no	IV (T2NxM1a)	>18	>18	>16	>16
84		M	74	unknown primitive			no	III				
85	lymph node metastasis	F	46	1.4	III	no	no	IV (T2aN1bM1d)	1	11	8	10
86	lymph node metastasis	F	80	unknown primitive			no	IV (T0NxM1a)	35	35	35	35
87	cutaneous metastasis	F	83	1.288	IV	no	surgery, immunotherapy	IV (T2aN1bM1a)	15	>80	7	>57
88	sub-cutaneous metastasis	M	70	0.241	II	no	surgery, immunotherapy	IV (T1aN3bM1c)	21	112	1	12
89	metastasis	M	54	2.831	III	no	surgery	IV (T3aN0M1c)	21	46	3	24
90	lymph node metastasis	M	76	4.95		yes	surgery, targeted therapy	IV (T4bN1bM1b)	30	>62	>11	>11
91	lymph node metastasis	F	71	8.3	III	yes	surgery	IV (T4bN1bM1b)	12	>43	11	>42
92	lymph node metastasis	F	50	4	IV		surgery	IIIC (T4N1bM0)	130	>140	0	>9
93	metastasis	M	64	2.1	III	no	surgery	IIIB (T3aN1aM0)	20	>65	2	>44
94	lymph node metastasis	F	59	20	IV	yes	no	IV (T4bN2bM1d)	>7	>7	>7	>7
95	sub-cutaneaous metastasis	M	61	NA			surgery	IV (TxN0M1c)	12	68	16	29
96	cutaneous metastasis	M	79	12	V	no	no	IV (T4aN1bM1c)	5	6	4	5
97	primary tumor	F	46	unknown		yes	surgery	IIIB (TxN1bM0)	>8	>8	>6	>6

Supplementary Table 3: Results of the Log-Rank tests run between iNKT cell frequencies and patient's clinical data

	blood				tumor			
	from diagnosis time		from sampling time		from diagnosis time		from sampling time	
	PFS	OS	PFS	OS	PFS	OS	PFS	OS
% iNKT within CD45	0.529	0.715	0.781	0.438	0.387	0.023	0.655	0.100
% iNKT within CD3	0.903	0.316	0.654	0.887	0.369	0.004	0.591	0.029

Supplementary Table 4: Results of the Log-Rank tests run between phenotypic features of iNKT cells and patient's clinical data

		blood				tumor			
		from diagnosis time		from sampling time		from diagnosis time		from sampling time	
		PFS	OS	PFS	OS	PFS	OS	PFS	OS
activation markers	CD40	0.498	0.456	0.429	0.154	0.952	0.546	0.778	0.454
	CD69	0.991	0.797	0.898	0.790	0.037	0.515	0.436	0.885
	CD86	0.614	0.857	0.436	0.452	0.313	0.822	0.009	0.466
	CD25	0.763	0.233	0.525	0.328	0.821	0.113	0.947	0.480
NCR	NKG2A	0.896	0.834	0.692	0.815	0.933	0.468	0.444	0.710
	NKG2C	0.976	0.536	0.593	0.558	0.114	0.108	0.074	0.362
	NKG2D	0.896	0.961	0.692	0.995	0.848	0.567	0.876	0.793
	NKp30	0.480	0.204	0.358	0.299	0.814	0.501	0.334	0.288
	NKp44	0.931	0.796	0.923	0.701	0.759	0.561	0.474	0.850
	NKp46	0.949	0.245	0.474	0.459	0.294	0.806	0.001	0.914
ICP-ligands	OX40L	0.767	0.903	0.704	0.667	0.201	0.777	0.045	0.835
	ICOSL	0.758	0.796	0.417	0.619	0.767	0.364	0.750	0.274
	PDL1	0.490	0.027	0.570	0.027	0.621	0.582	0.757	0.679
	PDL2	0.320	0.668	0.445	0.519	0.404	0.302	0.560	0.695
	4.1BBL	0.453	0.939	0.476	0.495	0.478	0.895	0.321	0.725
ICP	4.1BB	0.955	0.595	0.804	0.371	0.208	0.182	0.742	0.740
	PD1	0.951	0.384	0.630	0.672	0.311	0.484	0.385	0.623
	OX40	0.385	0.571	0.720	0.543	0.970	0.779	0.736	0.358
	ICOS	0.453	0.939	0.417	0.265	0.244	0.440	0.543	0.751
	TIM3	0.175	0.748	0.814	0.296	0.261	0.223	0.067	0.053
	CTLA4	0.993	0.660	0.247	0.379	0.295	0.021	0.413	0.037
	LAG3	0.595	0.618	0.093	0.270	0.640	0.029	0.278	0.001
differentiation stage	CD27- CD45RA+ (EMRA)	0.758	0.971	0.535	0.891	0.639	0.612	0.980	0.640
	CD27+ CD45RA+ (N)	0.296	0.734	0.731	0.466	0.639	0.612	0.980	0.640
	CD27- CD45RA- (EM)	0.011	0.857	0.022	0.273	0.807	0.590	0.630	0.703
	CD27+ CD45RA- (CM)	0.741	0.939	0.666	0.995	0.817	0.800	0.544	0.886
CD56	CD56	0.908	0.690	0.834	0.718	0.782	0.586	0.879	0.263
iNKTreg	Treg (CD25+FoxP3+)	0.850	0.571	0.908	0.722	0.828	0.341	0.587	0.602

Supplementary Table 5: Results of the Log-Rank tests run between metabolism parameters and patient's clinical data

	blood				tumor			
	from diagnosis time		from sampling time		from diagnosis time		from sampling time	
	PFS	OS	PFS	OS	PFS	OS	PFS	OS
global metabolism (MFI puro)	0.016	0.497	0.051	0.412	0.035	0.457	0.531	0.916
Glucose dependency	0.499	0.320	0.856	0.320	0.147	0.383	0.029	0.383
Mitochondrial dependency	0.809	0.169	0.856	0.169	0.809	0.988	0.031	0.988
Glycolytic capacity	0.499	0.508	0.856	0.508	0.276	0.757	0.842	0.757
FAO&AAO capacity	0.809	0.320	0.982	0.320	0.073	0.988	0.089	0.988

Supplementary Figure 1

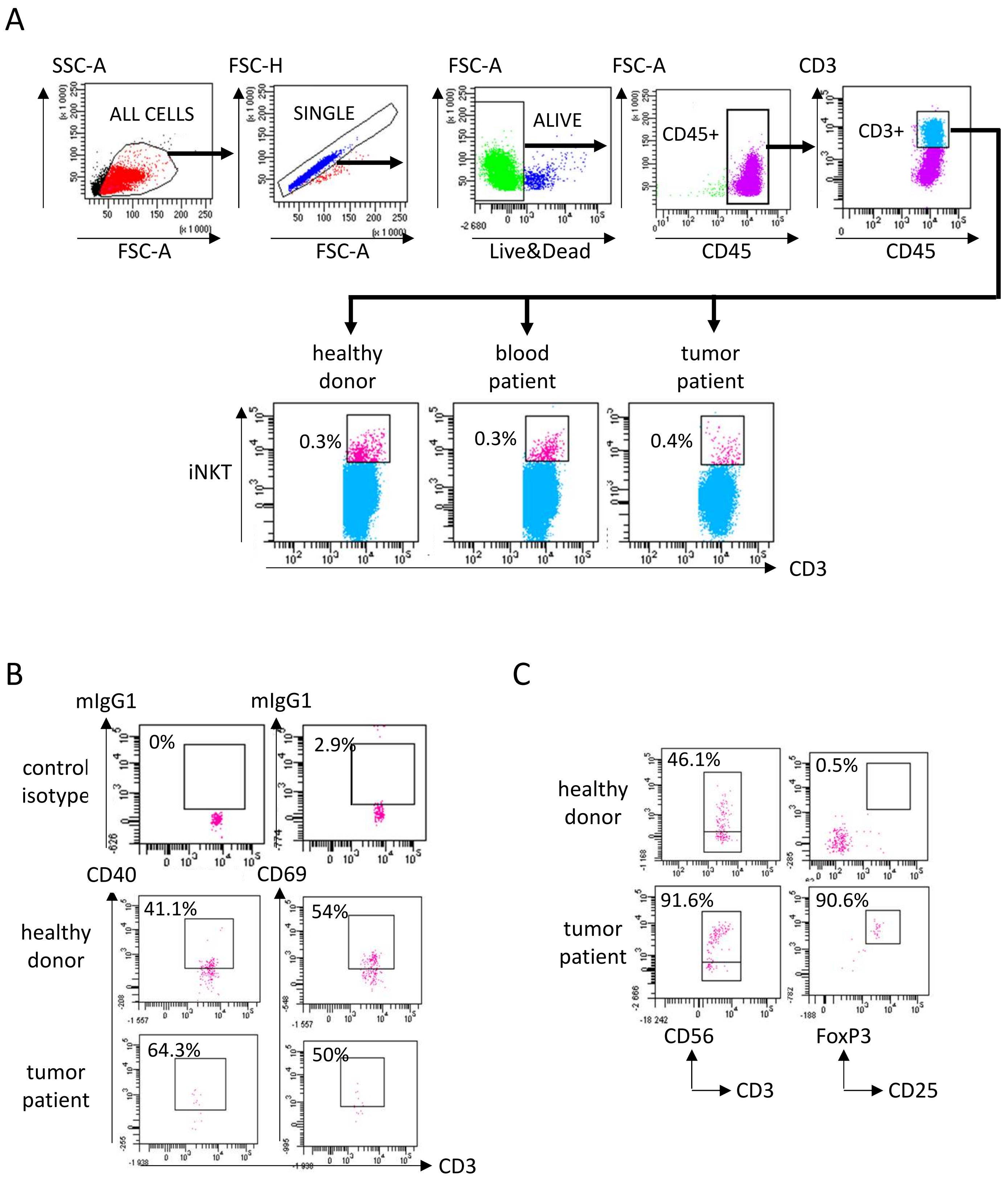

Supplementary Figure 1 (follow)

D

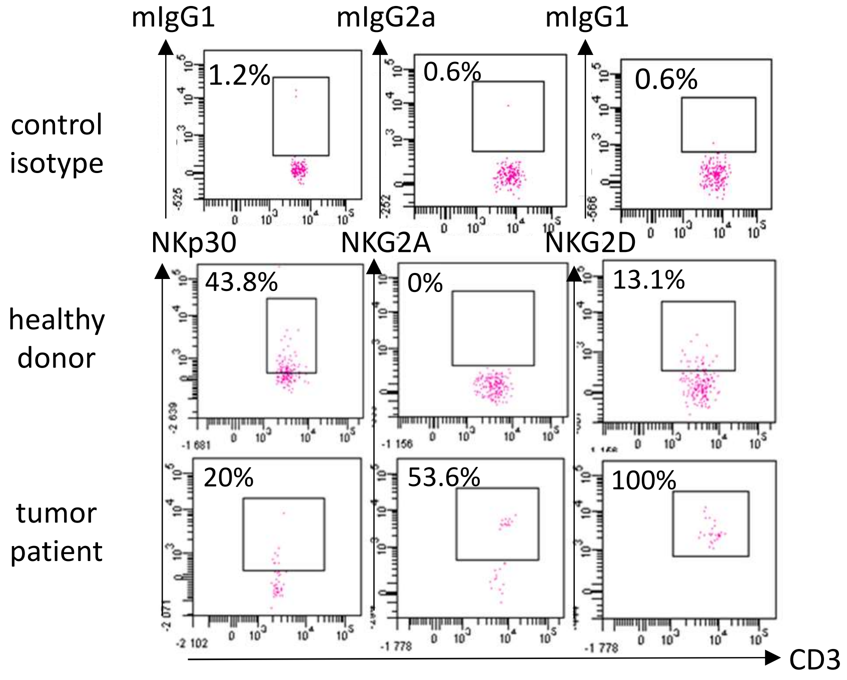

E

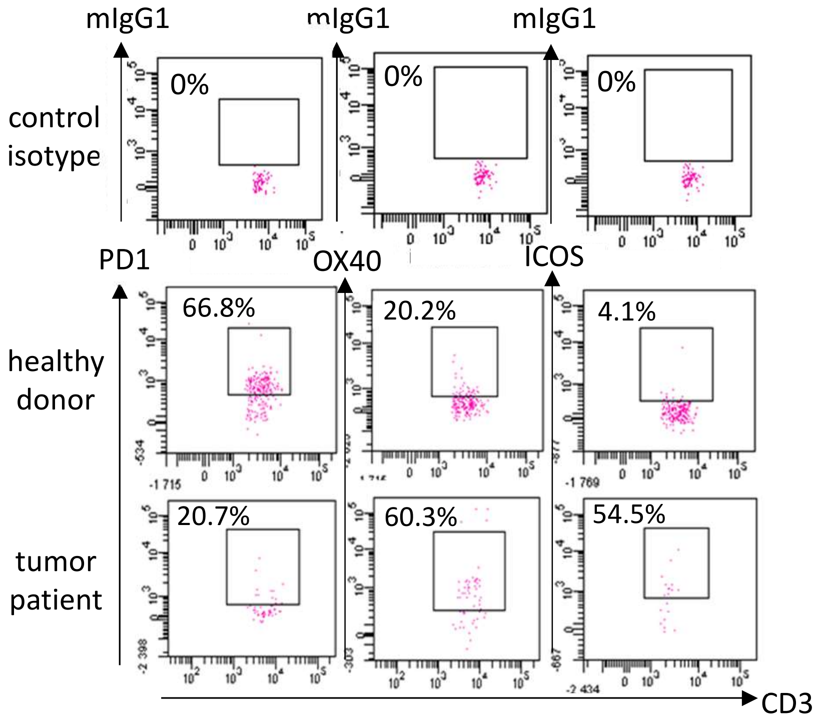

F

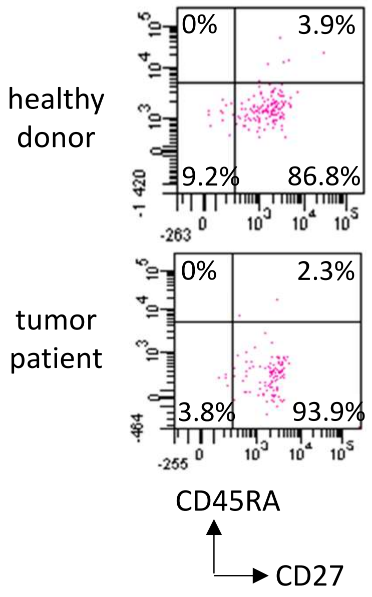

Supplementary Figure 2

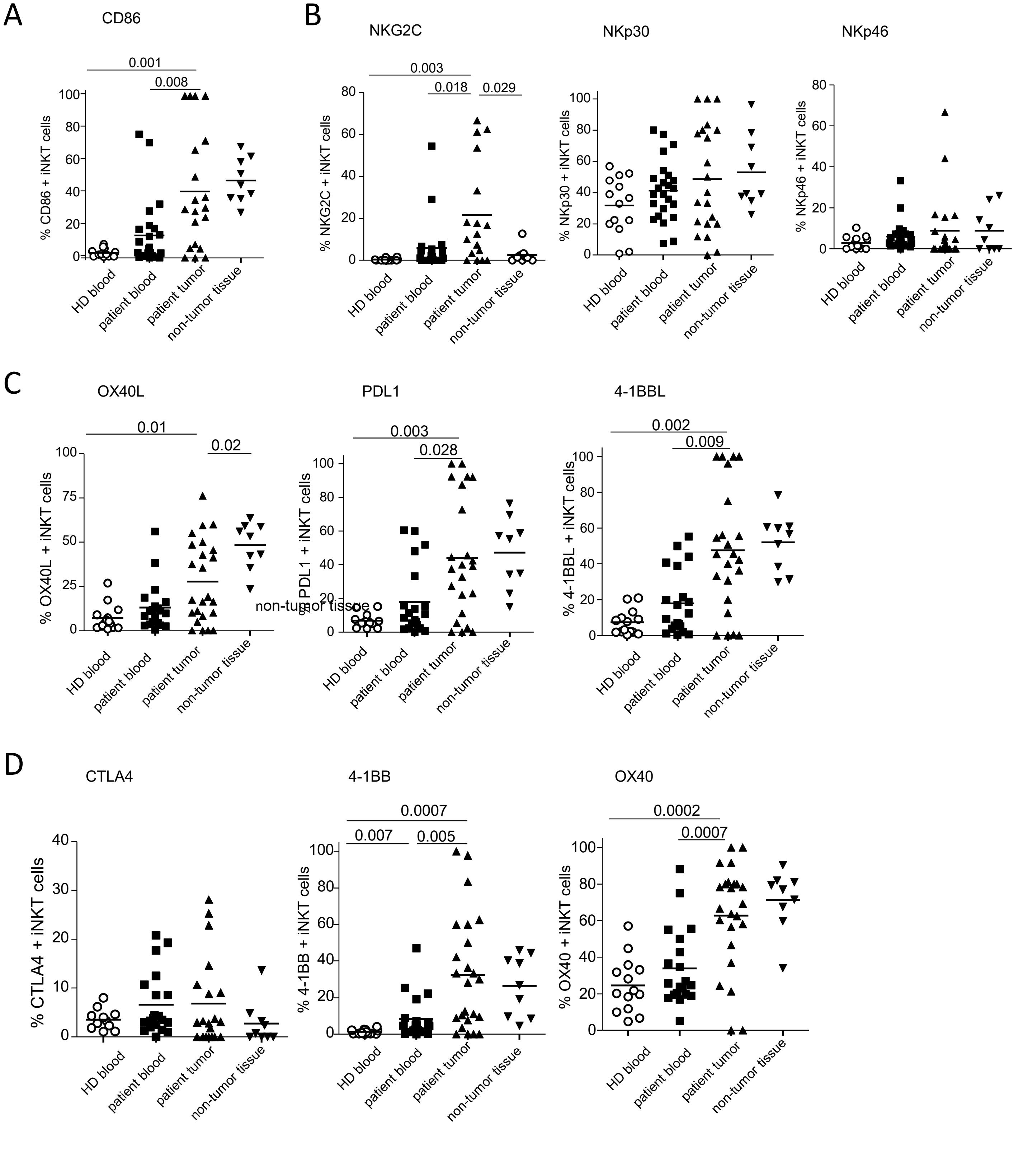

Supplementary Figure 2 (follow)

E

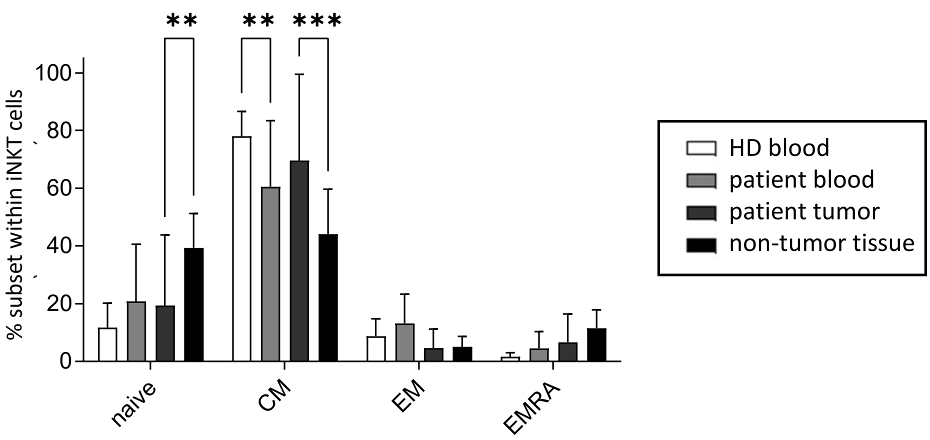

F

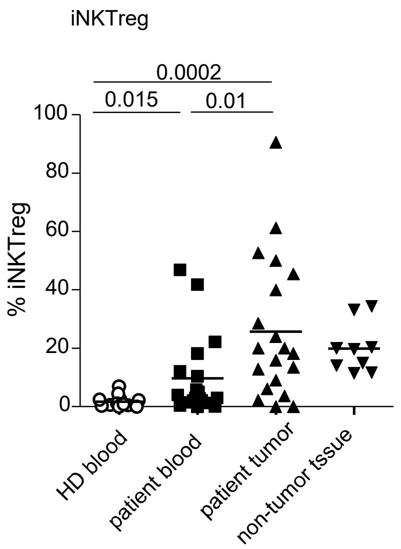

Supplementary Figure 3

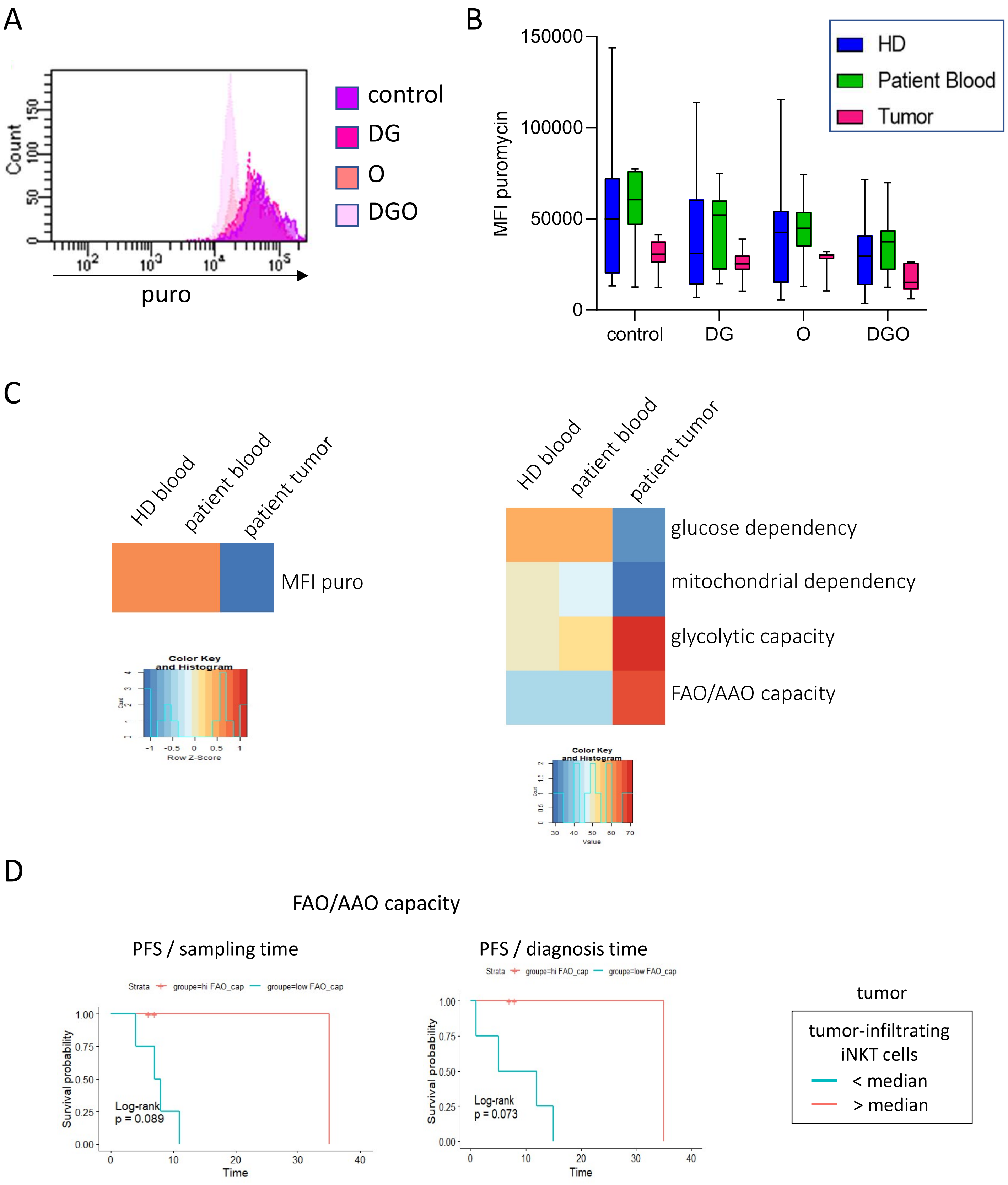

Supplement: Supplementary file 1 [file DataSheet1.pdf]
